# Estrogen receptor *α* regulates non-canonical autophagy that provides stress resistance to neuroblastoma and breast cancer cells and involves BAG3 function

**DOI:** 10.1038/cddis.2015.181

**Published:** 2015-07-09

**Authors:** V Felzen, C Hiebel, I Koziollek-Drechsler, S Reißig, U Wolfrum, D Kögel, C Brandts, C Behl, T Morawe

**Affiliations:** 1 Institute of Pathobiochemistry, University Medical Center of the Johannes Gutenberg University, Mainz, Germany; 2 Institute of Molecular Medicine, University Medical Center of the Johannes Gutenberg University, Mainz, Germany; 3 Department of Cell and Matrix Biology, Institute of Zoology, Johannes Gutenberg University Mainz, Mainz, Germany; 4 Experimental Neurosurgery, Neuroscience Center, Goethe University Hospital, Frankfurt, Germany; 5 Department of Medicine, Hematology/Oncology and University Cancer Center Frankfurt, Goethe University Hospital, Frankfurt, Germany

## Abstract

Breast cancer is a heterogeneous disease and approximately 70% of newly diagnosed breast cancers are estrogen receptor (ER) positive. Out of the two ER types, *α* and *β*, ER*α* is the only ER that is detectable by immunohistochemistry in breast cancer biopsies and is the predominant subtype expressed in breast tumor tissue. ER-positive tumors are currently treated with anti-hormone therapy to inhibit ER signaling. It is well known that breast cancer cells can develop endocrine resistance and resistance to anti-hormone therapy and this can be facilitated *via* the autophagy pathway, but so far the description of a detailed autophagy expression profile of ER-positive cancer cells is missing. In the present study, we characterized tumor cell lines ectopically expressing ER*α* or ER*β* as well as the breast cancer-derived MCF-7 cell line endogenously expressing ER*α* but being ER*β* negative. We could show that ER*α*-expressing cells have a higher autophagic activity than cells expressing ER*β* and cells lacking ER expression. Additionally, for autophagy-related gene expression we describe an ER*α*-specific ‘*autophagy-footprint*' that is fundamentally different to tumor cells expressing ER*β* or lacking ER expression. This newly described ER*α*-mediated and estrogen response element (ERE)-independent non-canonical autophagy pathway, which involves the function of the co-chaperone Bcl2-associated athanogene 3 (BAG3), is independent of classical mammalian target of rapamycin (mTOR) and phosphatidylinositol 3 kinase (PI3K) signaling networks and provides stress resistance in our model systems. Altogether, our study uncovers a novel non-canonical autophagy pathway that might be an interesting target for personalized medicine and treatment of ER*α*-positive breast cancer cells that do not respond to anti-hormone therapy and classical autophagy inhibitors.

Macroautophagy (hereafter referred to as autophagy) is a highly conserved catabolic process and involved in various cellular functions. It occurs under physiological conditions at basal level and has a role in cell homeostasis by degrading misfolded as well as long-lived proteins and damaged organelles. Material to be degraded gets sequestered in vesicles known as autophagosomes that ultimately fuse with lysosomes. Autophagy is differentially regulated in aging and is also involved in pathophysiological processes including cancer.^1, 2, 3^ Canonical autophagy responds to environmental cues *via* a variety of factors that mainly belong to homologs of autophagy-related (atg) genes originally identified in yeast.^4^ The two major regulators controling canonical autophagy are the mammalian target of rapamycin (mTOR) complex 1 (mTORC1) that negatively regulates autophagic activity and the Beclin1/class III phosphatidylinositol 3-kinase (PI3K) complex required for nucleation of the autophagosomal membrane. Membrane expansion is carried out by two ubiquitin-like conjugating systems (ATG12-ATG5 and ATG8/LC3) and the ATG18 protein family members WD repeat domain phosphoinositide interacting 1-3 (WIPI1-3) (autophagy regulation is excellently reviewed in refs. 5, 6, 7).

However, recently non-canonical autophagy pathways were discovered that differ from canonical signaling, as they do not necessarily require the hierarchical action of the ATG proteins and protein complexes.^8^ For example, Beclin1-independent or mTORC1- and ULK1 complex-bypassing non-canonical autophagy routes are known^9, 10, 11, 12^ but they finally all lead to fusion of autophagosomes with lysosomes and degradation of substrates in these acidic compartments.

In line with the complexity of the so far described autophagy pathways are numerous implications of autophagy in pathophysiological processes. For example, early tumorigenesis and tumor maintenance as well as the effectiveness of therapeutic intervention are affected by autophagy.^13, 14, 15, 16^ Altered ATG protein expression and altered autophagic activity have been shown in different cancer tissues ranging from glioblastoma stem cells to breast cancer cells. Recently, the co-chaperone Bcl-2-associated athanogene 3 (BAG3) that modulates age-related autophagic activity was shown to diminish proteotoxicity *via* selective autophagy and is highly expressed in estrogen receptor-positive neuroblastoma and breast cancer cells.^17, 18, 19, 20, 21^

Breast cancer is one most leading cause of cancer-related death in women. A great effort is ongoing to develop new strategies for treating its various forms subdivided into three classes: (I) hormone receptor-positive breast cancers that display approximately 70–80% of all cases, (II) human epidermal growth factor receptor 2 (HER2) overexpressing cancers in approximately 10–15% of all cases and (III) the remaining 10–15% of breast cancers that are defined by hormone receptor and HER2 negativity.^22, 23, 24^ Estrogen receptor (ER)-positive breast cancers show expression of two structurally related receptors ER*α* and ER*β*. Of these, ER*α* is the predominant subtype expressed in breast tumor tissue as it primarily stimulates cancer cell growth.^25^ Both receptors bind estrogen (17*β*-estradiol, E2) as ligand and patients with ER-positive tumors are currently treated with anti-hormone therapy utilizing either anti-estrogens (AE) interfering with ER signaling directly or blocking E2 synthesis with aromatase inhibitors. However, almost 40% of ER-positive breast cancers fail to respond to AE and tumor cells often develop resistance to anti-hormone therapy.^26^ Despite much progress in understanding this disease the number of patients dying from breast cancer is not decreasing substantially. This emphasizes the need for alternative strategies or add-on concepts in treating breast cancer. Autophagy inhibitors sensitize cancer cells to anti-cancer therapy and, therefore, currently represent attractive therapeutic tools.^27^ Nevertheless, as breast cancer is a heterogeneous disease and acquired resistance to anti-hormone therapy and efficacy of autophagy inhibitors can individually vary personalized treatment concepts focusing on detailed description of the autophagy pathway is needed.

In the present study, we provide evidence that ER*α* triggers non-canonical autophagy independent of ligand binding and its ERE-mediated transcription factor activity in different established ER expressing cellular tumor models and human breast cancer tissue. We show that reducing autophagic activity by knockdown of BAG3 and blocking lysosomal degradation sensitizes ER*α*-positive cells to stress. The detailed analysis of the autophagy pathway shown here might open up new strategies in treating ER*α*-positive breast cancer cells that do not respond to AE- or aromatase inhibitor therapy.

## Results

### ER expression differentially regulates transcription of autophagy-related genes

ERs are either transcription factors interacting directly with estrogen response elements (EREs) and *via* interaction with other transcription factors or activate cytoplasmatic signaling cascades. As ER*α* and ER*β* are usually co-expressed, a differential analysis of the function of each receptor is experimentally challenging. We employed a well-characterized neuroblastoma cell line (SK-N-MC) lacking expression of ERs stably transfected with mock-plasmid (SK-01), estrogen receptor *α* (SK-ER*α*) or estrogen receptor *β* (SK-ER*β*).^28, 29, 30^ These cell lines enable us to study the differential function of ERs in tumor cells under controlled and well-described conditions.^21, 28, 30, 31, 32, 33, 34^ Additionally, we also employed the patient-derived MCF-7 breast cancer cell line as an ER*α*-positive breast cancer model endogenously expressing ER*α* but not ER*β*. All cell lines were characterized for ER expression by PCR analysis (Supplementary Figure 1a). As described previously, ER expression as well as enhanced autophagic activity contributes to metastatic potential and therapy resistance of different cancer types.^18, 35^

**Figure 1:**
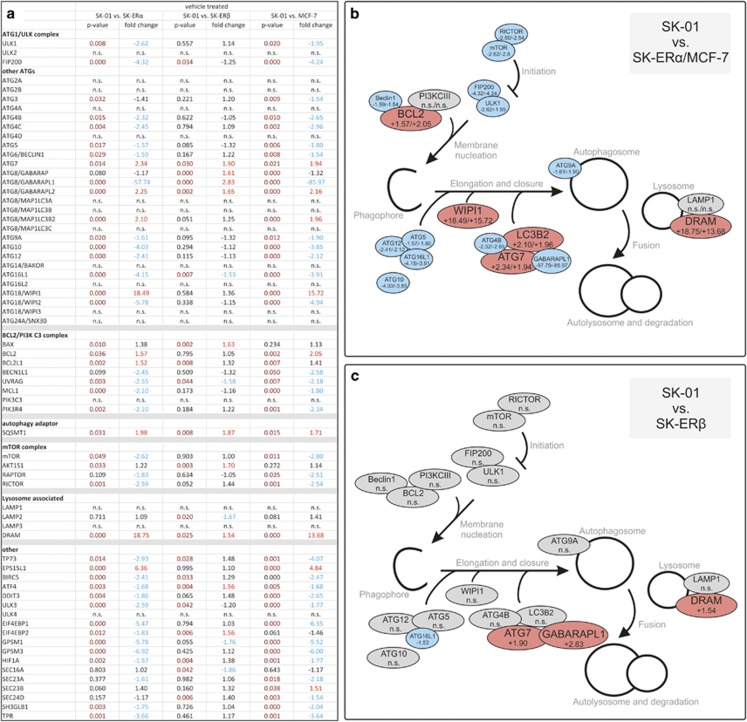
Estrogen receptors differentially regulate autophagy pathway-associated gene expression. (**a**) Total RNA from SK-01, SK-ER*α*, SK-ER*β* and MCF-7 cells was characterized by using the Human Autophagy Primer Library 1 (HATPL-1) and comparing ER expressing cells to mock-plasmid transfected controls (SK-01). Red numbers indicate an upregulation greater than 1.5 fold, blue numbers indicate an downregulation greater than 1.5 fold, dark red numbers denote a *P*-value of <0.05, black numbers depict no significant change and n.s. no significant change in gene expression within the whole group. Results represent the mean values of five independent experiments. (**b** and **c**) Cartoons display a selection of key autophagic genes of the corresponding PCR array analysis in (**a**) and show the ‘*autophagy-footprint*' of ER*α* (SK-ER*α* and MCF-7) and ER*β* expressing cells. Genes highlighted in blue were downregulated, in gray not altered in expression whereas genes highlighted in red were upregulated. First numbers in (**b**) show the regulation in SK-ER*α* cells and following numbers the regulation in MCF-7 cells. In (**c**) numbers display the regulation of genes in SK-ER*β* cells compares to SK-01. n.s. indicates that there is no significant change in gene expression comparing SK-ER*α*, SK-ER*β* and MCF-7 cells to mock-transfected controls

**Figure 2:**
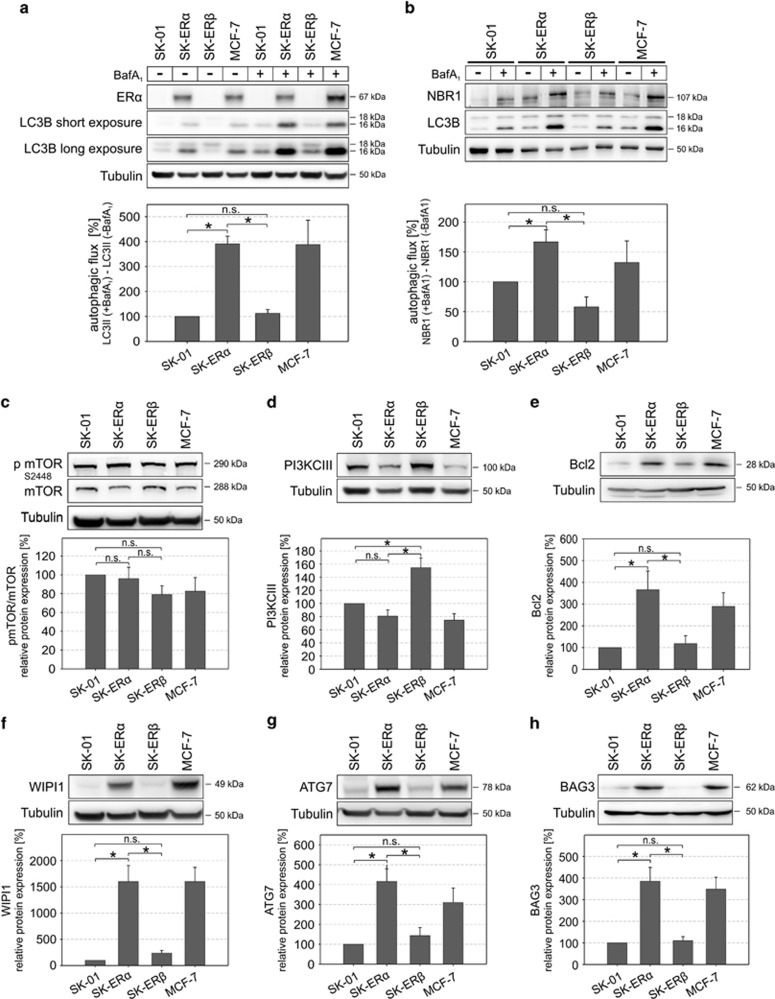
ER*α* expression enhances autophagic flux and differentially modulates key autophagy pathway-related protein expression. (**a** and **b**) Protein extracts from vehicle or BafA_1_ treated SK-01, SK-ER*α*, SK-ER*β* and MCF-7 cells were subjected to western blot analysis and calculation of autophagic flux using anti-LC3 and NBR1 antibodies. Tubulin was used as a loading control. (**a**) ER*α* expression of the different cell lines was shown *via* western blot analysis and autophagic flux was determined by the accumulation of LC3-II in a 6 h treatment period with 500 nM BafA_1_. Therefore, normalized LC3-II levels in the absence of the lysosomal inhibitor were subtracted from the corresponding levels obtained in the presence of BafA_1_. (**b**) Cells were treated as in (**a**) and autophagic flux was calculated by NBR1 expression determined by western blot. (**c**–**h**) Western blot analysis of protein extracts from untreated cells were performed for detection of indicated proteins. In the diagrams (lower panel each) levels of proteins are depicted after normalization to corresponding Tubulin levels. Values of three independent experiments in each panel are expressed as mean±S.E.M. and control SK-01 cells were set to 100%. (*) on bars represents statistical significance of *P*<0.05 comparing two groups and n.s. displays no statistical significant difference

To study the link between ER expression and autophagy, we first performed gene expression profiling of the different cell types employing a human autophagy PCR array. Utilizing SK-01 cells as control cells not expressing ERs, we calculated gene expression of cells ectopically or endogenously expressing ER*α* or ER*β*. We analyzed groups of genes displaying the different regulatory complexes, various other ATGs, that are associated with lysosomal function and candidates that are not part of the core autophagy machinery. The results of the PCR analysis showed reduced expression of multiple genes and key autophagy components in ER*α*-expressing cells although we could detect higher autophagic activity in these cells (Figures 1a, 2a and b), whereas also an upregulation for nine genes was shown. In contrast, SK-ER*β* cells showed a negative regulation for 4 genes and an upregulation for 10 genes that was not accompanied by higher autophagic activity and these genes were mainly different to those regulated in ER*α*-expressing cells. From these data, we established an ‘*autophagy-footprint*' of cells expressing ER*α* or ER*β* (Figures 1b and c) and showed a fundamental difference in autophagy-related gene expression. Additionally, we could demonstrate that SK-ER*α* cells that ectopically express ER*α* have the almost identical ‘*autophagy-footprint*' as ER*α*-positive MCF-7 breast cancer cells. Taken together, these data demonstrate that ER expression differentially regulates autophagy-related gene transcription in tumor cells.

### ER*α* induces non-canonical autophagic activity

Since we observed differential regulation of autophagy-related genes in ER*α*-expressing cells *versus* SK-ER*β* or SK-01 cells, we further investigated whether SK-ER*α* and MCF-7 cells show an altered autophagic activity. We first performed western blotting for LC3B (hereafter referred to as LC3), a widely accepted autophagosome marker. In ER*α*-expressing cells, the accumulation of LC3-II as well as NBR1, another well-established marker for autophagic degradation, was significantly higher than in mock-transfected SK-01 or ER*β*-expressing cells (Figures 2a and b).^36^ To verify that the observed effects are not clonal artifacts, we screened two different stable clones of each cell type for autophagic activity by western blotting for LC3 (Supplementary Figure 1b) and observed no difference in autophagic activity comparing the subclones.

Next, we determined the effect of ERs on expression of the major protein complexes of initiation, nucleation, elongation and closure of autophagosomal membranes. In most eukaryotic cellular systems, mTORC1 has been demonstrated to negatively regulate the onset of autophagy and activated mTOR is phosphorylated at serine 2448.^37, 38^ Although we showed that ER*α*-positive cells display an enhanced autophagic flux we observed no significant changes in the mTOR to phospho mTOR ratio (Figure 2c). In line with the mTOR data, we could also not detect an altered ratio of p70S6K to phospho p70S6K, (Supplementary Figure 2), a downstream target of mTOR kinase. Surprisingly PI3KCIII, which positively controls autophagosome formation, is not upregulated in ER*α*-expressing cells (Figure 2d). PI3KCIII activity is regulated by Beclin1, which itself is bound and inhibited by Bcl2.^39^ Figure 2e shows that ER*α*-positive cells express Bcl2 at higher levels than SK-01 cells or the SK-ER*β* clone, according to PCR array data. Further we could not reverse the autophagic flux in the ER*α*-expressing cells with knockdown of Beclin1, a key factor for canonical autophagy,^40^ suggesting that a Beclin1-independent non-canonical autophagic pathway exists in our cell model (Supplementary Figure 3). Additionally, we inhibited the activity of the PI3K complex with the specific PI3K inhibitor Wortmannin (WN) and again we could not detect a decreased autophagic flux in ER*α*-expressing cells (Supplementary Figure 4a).

Consecutively, we investigated the protein level of effectors downstream of the major autophagy regulating protein complexes. We showed a strong upregulation of WIPI1 and ATG7 in ER*α*-positive cells compared with SK-01 and SK-ER*β* cells (Figures 2f and g), which have been identified earlier to function downstream of PtdIns(3)P mediating events during initiation of autophagosome formation.^41^ BAG3 was already described to modulate autophagy in aged cells, to mitigate proteotoxicity *via* selective autophagy and non-canonical autophagy induced by proteasome inhibition.^2, 17, 32^ Compared with controls, BAG3 expression in SK-ER*α* and MCF-7 was strongly enhanced and unaltered in SK-ER*β* cells (Figure 2h). It has been shown that components of the Mitogen-activated protein kinase (MAPK) pathway together with the autophagy-related proteins DRAM1 and SQSTM1 lead to enhanced autophagic activity controling migration/invasion in cancer stem cells.^18^ As we showed increased DRAM1 and SQSTM1 mRNA, we asked whether blocking MAPK pathway activity in ER*α*-positive cells would result in lowered autophagic flux. We could not detect reduced autophagic flux after U0126 and subsequent BafA_1_ treatment (Supplementary Figure 4b). Interestingly, DRAM1 is partially contributing to this specific ER*α*-driven autophagic pathway, as we detected a reduced autophagic flux in ER*α*-expressing cells after DRAM1 knockdown (Supplementary Figure 5a).

Additionally, we also investigated whether reducing ER*α* levels in MCF-7 cells changes autophagic activity but knockdown of ER*α* did not significantly alter the autophagic flux or BAG3 expression level. Obviously, the residual amount of ER*α* expression after RNAi knockdown is still sufficient to fulfill its role in enhancing non-canonical autophagy (Supplementary Figure 5b). This interpretation is supported by the observations that transiently overexpressing ER*α* in ER-lacking native SK-N-MC cells (precursors of SK-01, SK-ER*α* and SK-ER*β* cells) leads to enhanced autophagic flux and elevated BAG3 protein levels (Supplementary Figure 5b).

**Figure 3:**
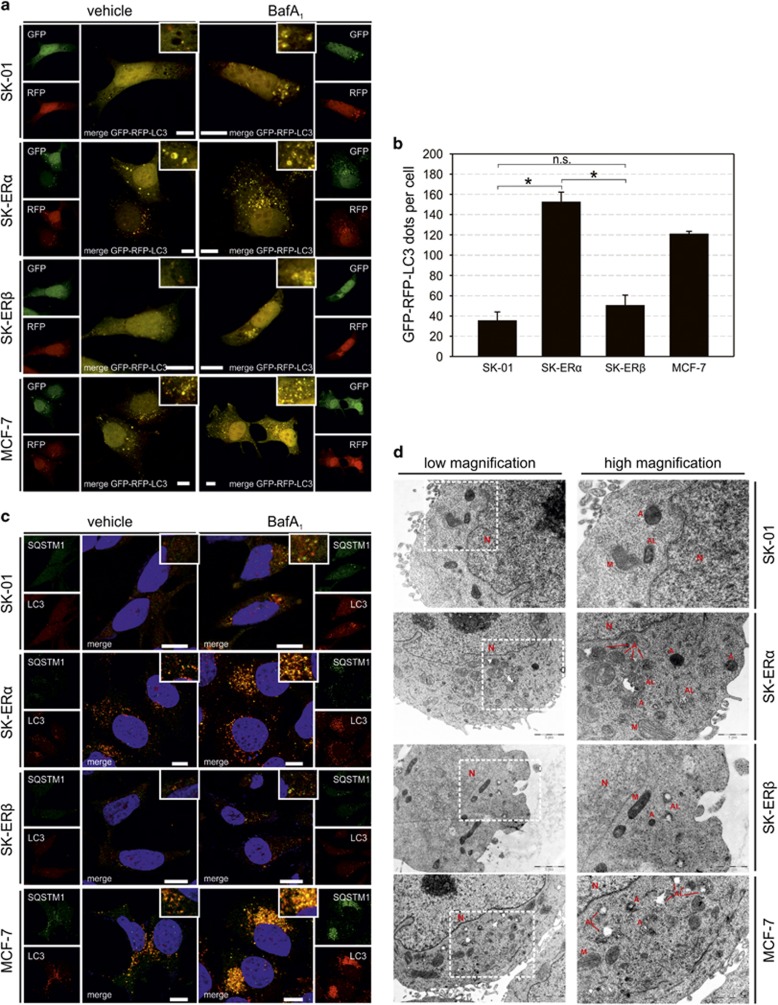
ER*α* expression promotes an increase of autophagosomes and autophagic flux. (**a**) SK-01, SK-ER*α*, SK-ER*β* and MCF-7 cells were transiently transfected with a GFP-RFP-LC3 fusion protein and treated with vehicle control and 500 nM of BafA_1_ for 6 h. In autophagosomes, both RFP and GFP signals are apparent whereas under acidic conditions when autophagosomes fuse with lysosomes just the RFP signal persists. In contrast, GFP signal diminishes due to a labile enhanced GFP under acidification. Representative images acquired by confocal microscopy are shown. Scale bars: 10 *μ*m. (**b**) GFP-RFP-LC3-punctae were counted in all cell lines transfected with the ptfLC3 expression plasmid and treated with 500 nM of BafA1 for 6 h before fixation. At least six different positions of the corresponding stainings per experiment were documented utilizing confocal laser scanning microscopy and counted numbers of GFP-RFP-LC3 punctae were averaged. 59 SK-0 cells, 56 SK-ER*α* cells, 59 SK-ER*β* cells and 58 MCF-7 cells were analyzed. Three independent experiments in each panel are expressed as mean±S.E.M. (*) on bars represents statistical significance of *P*<0.05 comparing two groups and n.s. displays no statistical significant difference. (**c**) Representative pictures of indirect immunofluorescence staining of endogenous LC3 (red) and SQSTM1 (green) in SK-01, SK-ER*α*, SK-ER*β* and MCF-7 cells, treated with vehicle control and 500 nM of BafA_1_ for 6 h, are shown. DAPI (blue) was used to stain DNA. Scale bars: 10 μm. (**d**) Typical ultrastructure of SK-01, SK-ER*α*, SK-ER*β* and MCF-7 cells visualized by using transmission electron microscopy. Magnifications of marked areas are shown in the panels to the right. ER*α*-expressing cells showed increased autophagic vesicles of different stages of maturation. Autophagosomes are indicated by A, autolysosomes by AL, nucleus by N and mitochondria by M

To corroborate that our finding of increased expression of autophagic markers in ER*α* cells reflect an enhanced autophagosome biogenesis rather than a decreased autophagosome clearance, we used an expression vector (ptfLC3) encoding LC3 fused to red fluorescent protein (RFP) and green fluorescent protein (GFP) in tandem (GFP-RFP-LC3).^42^ We showed that all cell lines display red fluorescent punctae with no corresponding signal in the green channel under control conditions indicating intact fusion of autophagosomes with lysosomes. Consistent with western blotting results in Figures 2a and b, we observed an enhanced accumulation of autophagosomes in ER*α*-positive cells (Figure 3a). Quantification of GFP-RFP-LC3 punctae in every cell line (Figure 3b) further underlines our findings and demonstrates significantly higher amounts of autophagolysosome formation in ER*α*-expressing cells.

Additionally, immunofluorescence stainings of endogenous LC3 and SQSTM1 clearly emphasize an enhanced accumulation after BafA_1_ treatment in SK-ER*α* and MCF-7 cells (Figure 3c). Identifying the presence of autophagic vesicles by transmission electron microscopy is considered as gold standard of verifying autophagic activity.^43^ Indeed, an abundance of autophagosomes and autophagolysosomes was detected in ER*α*-expressing cells compared with SK-01 and SK-ER*β* cells (Figure 3d).

These results suggest that ER*α* expression leads to enhanced autophagic flux, which is not controlled by the mTOR, PI3KCIII and MAPK pathway but key autophagy proteins such as BAG3, ATG7 and WIPI1 are strongly upregulated. This newly described non-canonical autophagy pathway is driven by ER*α*.

### Increased autophagic flux is independent of ERE-mediated transcriptional activity of ER*α*

**Figure 4:**
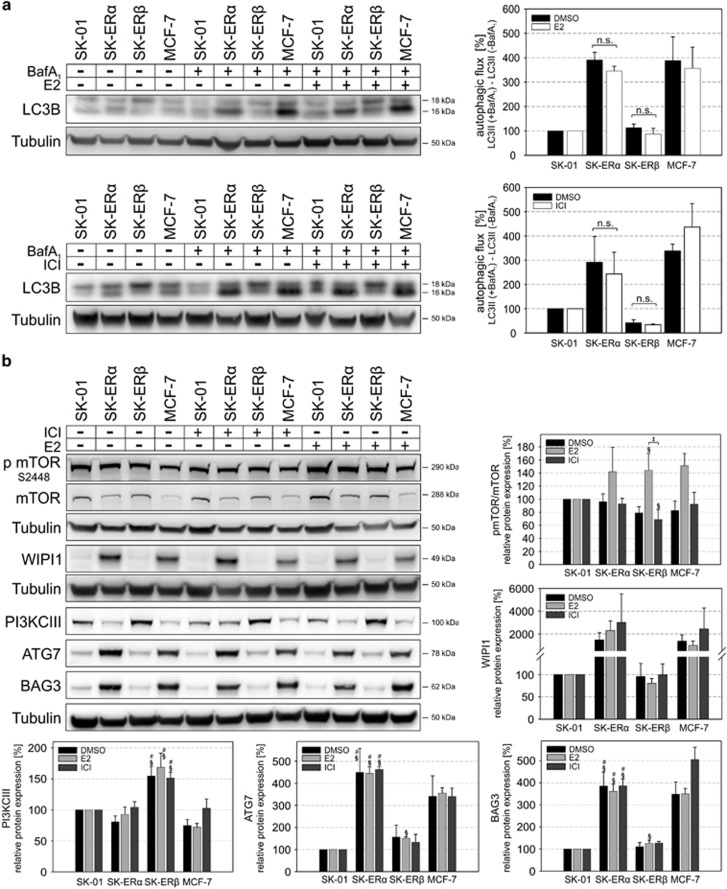
E2 or ICI treatment does not alter autophagic flux or expression of key autophagy pathway-related proteins. (**a**) SK-01, SK-ER*α*, SK-ER*β* and MCF-7 cells were incubated with 10 nM E2 and 1 *μ*M ICI or vehicle control for 24 h. Six hours before cell lysis indicated cells were additionally treated with 500 nM BafA_1_ and subjected to western blot analysis. Autophagic flux was determined by the accumulation of LC3-II. Therefore, normalized LC3-II levels in the absence of the lysosomal inhibitor were subtracted from the corresponding levels obtained in the presence of BafA_1_. Tubulin was used as a loading control. E2 or ICI treatment does not affect autophagic flux in each of the analyzed cell types. (**b**) Cells were treated with E2 or ICI for 24 h and western blot analysis of indicated proteins was performed. Tubulin served as a loading control. Except of the pmTOR/mTOR ratio of E2 and ICI treatment of SK-ER*β* there was no effect on protein expression of pmTOR/mTOR, WIPI1, PI3KCIII, ATG7 and BAG3 after drug treatment. (**a**, **b**) Values of threre independent experiments in each panel are expressed as mean±S.E.M. and control SK-01 cells were set to 100%. (§) on bars represents statistical significance of *P*<0.05 comparing SK-ER*α* or SK-ER*β* cells with SK-01 cells with the same treatment. (#) on bars represents statistical significance of *P*<0.05 comparing E2- or ICI-treated SK-ER*α* cells with E2- or ICI-treated SK-ER*β* cells. (‡) on bars displays a statistical significance of *P*<0.05 of E2-treated SK-ER*β* cells compared with ICI-treated SK-ER*β* cells. All other combinations show no statistical significant difference

ER-mediated transcription factor activity can be induced *via* its ligand E2 or inhibited *via* the synthetic antagonist ICI 182780 (ICI). We treated all cell lines with E2 and ICI and measured expression of autophagy-related genes according to Figure 1. The cell lines tested showed classical ER signaling as we could induce ERE-mediated transcription factor activity *via* E2 and also reduce the ERE-Luciferase signal after ICI treatment (Supplementary Figure 6). Interestingly, neither major changes in autophagy-related gene expression (Supplementary Figure 7) nor changes in autophagic activity or expression of key autophagy proteins in any of the analyzed cells after respective drug application were observed (Figures 4a, b and 2a). The key autophagy proteins studied in Figures 2c, d and 2f–h also showed no significant changes in total protein expression and the ratio between phosphorylated and total mTOR was not altered (Figure 4b).

The data provided here indicate that ER*α*-mediated enhanced autophagic activity is independent of its ERE-mediated transcription factor activity and the observed ER-driven non-canonical autophagy pathway is fundamentally different to classical canonical autophagy.

### Inhibition of autophagy reduces resistance of ER*α*-expressing cells to oxidative stress

**Figure 5:**
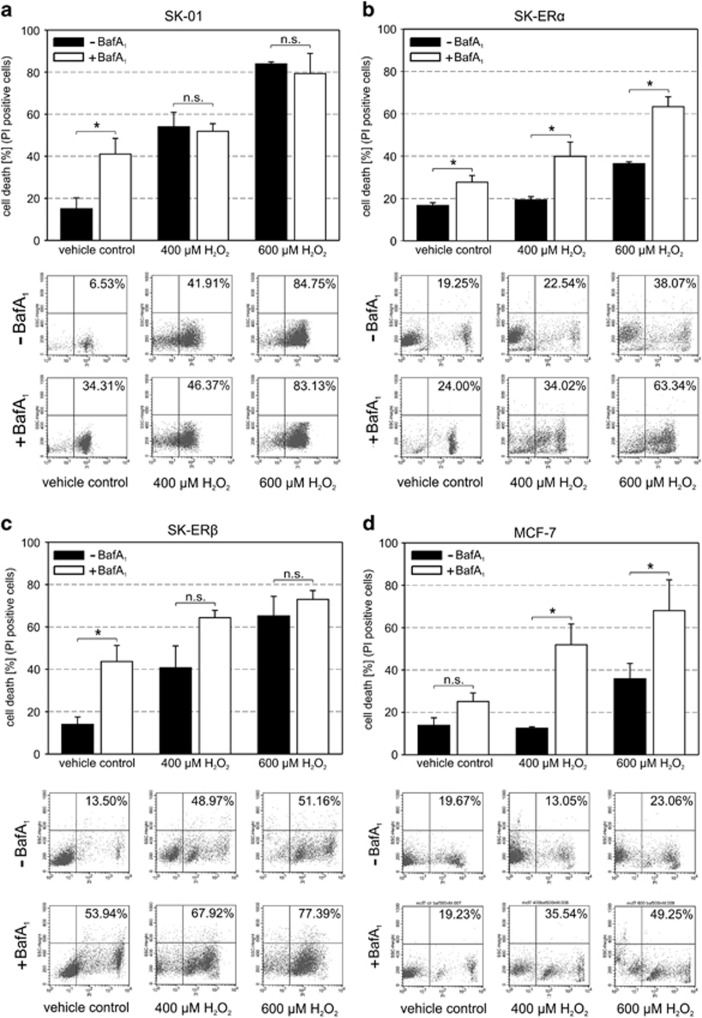
Inhibiting the late phase of autophagy *via* BafA_1_ results in enhanced cell death in ER*α-*expressing cells upon exposure to oxidative stress induced by H_2_O_2_. SK-01, SK-ER*α*, SK-ER*β* and MCF-7 cells were treated with vehicle control or 400 *μ*M and 600 *μ*M H_2_O_2_ with or without BafA_1_ (500 nM) for 24 h and cell death was quantified by flow cytometry after propidium iodid staining. Values of three independent experiments in each panel are expressed as mean±S.E.M. and representative data from the experiments are shown in FACS dot plot profiles. (*) on bars represents statistical significance of *P*<0.05 comparing two groups and n.s. displays no statistical significant difference

Oxidative stress can lead to preferential killing of cancer cells and various pharmacological compounds with direct or indirect effects on the induction of reactive oxygen species have been used in cancer therapy.^44^ Cells react on increased oxidative stress *via* the induction of the autophagy pathway to modify the cellular protein homeostasis. It is also discussed that autophagy is a key mechanism of cell survival during anti-estrogen treatment and progression of drug resistance in breast cancer cells.^45, 46^ We previously showed that ER*α* expression mediates increased resistance of neuroblastoma cells to oxidative stress.^21^ To further investigate whether ER*α*-induced autophagy contributes to increased cell viability after stress induction we co-treated the cell lines with BafA_1_ to inhibit the late phase of autophagy (Figures 5a–d). Interestingly, the inhibition of autophagy by BafA_1_ treatment in ER*α*-expressing cells significantly reduced cell survival after H_2_O_2_ treatment that was not observed in ER*α*-negative cells (Figures 5a–d), suggesting that enhanced autophagic activity mediated by ER*α* contributes to the resistance of cells to oxidative stress and drug treatment.

### Knockdown of BAG3 reduces autophagic flux and contributes to a partial reversal of stress resistance in ER*α*-expressing cells

**Figure 6:**
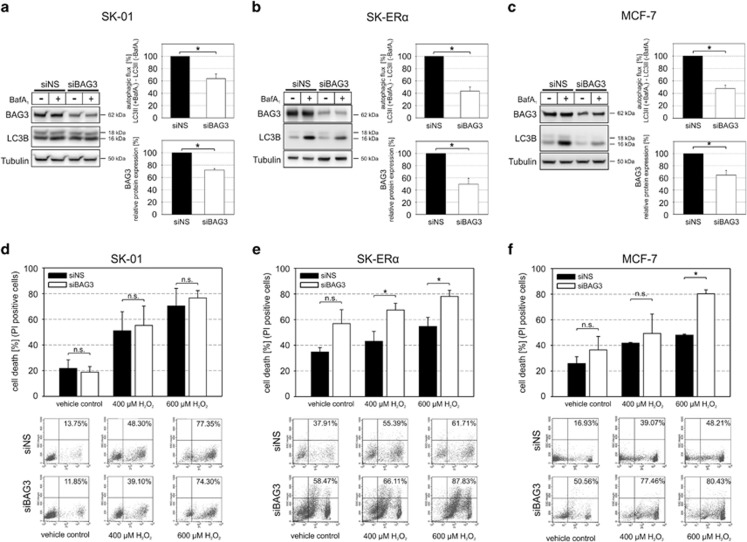
BAG3 is required for enhanced autophagic flux and resistance to oxidative stress in SK-ER*α* and MCF-7 cells. (**a**–**c**) SK-01, SK-ER*α* and MCF-7 cells were transfected with non-sense siRNA (siNS) and BAG3 siRNA (siBAG3) for 48 h, as indicated. Protein extracts from vehicle or BafA_1_-treated cells were subjected to western blot analysis and calculation of autophagic flux using anti-LC3 antibodies. Tubulin was used as a loading control. Autophagic flux was determined by the accumulation of LC3-II in a 6 h treatment period with 500 nM BafA_1_. Therefore, normalized LC3-II levels in the absence of the lysosomal inhibitor were subtracted from corresponding levels obtained in the presence of BafA_1_. (**d**–**f**) SK-01, SK-ER*α* and MCF-7 cells were transfected with non-sense siRNA (siNS) and BAG3 siRNA (siBAG3) for 48 h and treated with vehicle control or 400 *μ*M and 600 *μ*M H_2_O_2_ for 24 h, as indicated. Cell death was quantified by flow cytometry after propidium iodide staining. Values of three (SK-01), four (SK-ER*α*) and three (MCF-7) independent experiments in each panel are expressed as mean±S.E.M. and representative data from the experiments are shown in FACS dot plot profiles. (*) on bars represents statistical significance of *P*<0.05 comparing two groups and n.s. displays no statistical significant difference.

BAG3 has previously been shown to modulate selective autophagy, proteotoxicity, resistance to stress and cancer-related signaling networks and we have shown that BAG3 expression is upregulated in SK-ER*α* cells.^2, 17, 21, 47^ Knockdown of BAG3 was accompanied by reduced levels of LC3-II after lysosomal inhibition in ER*α*-positive cells and to a lesser extend in SK-01 cells, demonstrating that BAG3 downregulation significantly impairs autophagic flux (Figures 6a–c). Interestingly, upon siRNA-mediated BAG3 knockdown and H_2_O_2_ treatment we detected no changes of cell death in control cells, but a significant increase in sensitivity in ER*α*-expressing cells (Figures 6d–f), that was in total even higher than after inhibiting autophagy *via* BafA_1_ treatment (Figures 5b and d).

To translate our results obtained in cellular model systems to clinically relevant conditions, we analyzed tissue from breast cancer patients characterized to be ER positive or ER negative. We could show that overall the staining intensity of autophagy markers LC3 and SQSMT1 is higher in ER-positive compared with ER-negative breast cancer tissue (Supplementary Figure 8). BAG3 expression generally was increased in some ER-positive patients but interestingly in one case investigated (patient 17889) BAG3 protein levels were reduced in regions with high ER*α* expression (Supplementary Figure 8).

Together, this set of experiments shows that interfering with the autophagy pathway reduces the resistance of ER*α*-expressing cells and sensitizes them to cell death inducing stress. Additionally, in ER-positive breast cancer patients autophagy markers are strongly increased although individual changes in BAG3 expression occur.

## Discussion

Breast cancer is a heterogeneous disease and clinically classified as steroid hormone receptor-positive, HER2-positive and triple-negative tumors. Approximately 70% of newly diagnosed breast cancers are ER-positive. Out of the two forms of ERs, ER*α* levels are specifically analyzed in clinical specimens because estrogen binding to ER*α* primarily stimulates proliferation of breast cancer cells. It is well known that breast cancer cells can develop endocrine resistance and resistance to anti-hormone therapy and this can be facilitated by an upregulation of the autophagic machinery^48^ but so far the description of a detailed autophagy expression profile of ER-positive cancer cells is missing.

In our study, we employed a model of transformed cells ectopically expressing ER*α* or ER*β* as well as a patient-derived ER*α*-positive breast cancer cell line. We could show that the presence of ER*α* leads to a higher autophagic activity. This process facilitates stress resistance and survival after oxidative stress and is independent of the ERE-mediated transcription factor activity. We dissected the autophagy signaling pathway and could show that canonical key protein complexes were not specifically activated or enhanced in their expression. Surprisingly, other key autophagy-related genes out of the two ubiquitin-like conjugating systems were even markedly reduced in their expression in ER*α* cells. Furthermore, our analysis shows that treating ER*α*- or ER*β*-positive cells with E2 or ICI did not alter the identified ‘*autophagy-footprint*' of these cells suggesting an ER-modulated mechanism, which is different to their function acting as bona fide transcription factors. It has been shown that ERs also function in a non-classical mode by interacting with various intracellular signaling pathways, thereby affecting indirectly the transcription of target genes independently of the classical genomic action.^49, 50^ This may explain the observed ERE-independent effects of ER*α* on the autophagic pathway. Canonical autophagy involves the hierarchical activity of afore-mentioned signaling complexes and ATG proteins. However, data accumulate that the formation of functional autophagosomes can bypass some of these steps but the specific mechanisms of this non-canonical autophagy pathways are still under debate.^8, 9, 51^ Despite the downregulation of key autophagy genes, in our cellular system, WIPI1 and BAG3 are strongly expressed. Both proteins are key factors in regulating and executing selective autophagy and have been implicated in non-canonical autophagy pathways before.^2, 32, 51, 52^ Therefore, we provide evidence that this novel ER*α*-mediated non-canonical autophagy pathway described here, at least in part, is mediated by the function of BAG3. The partial reversal of stress resistance of ER*α*-expressing cells is seen after inhibiting the late phase of autophagy *via* BafA_1_ treatment as well as after knockdown of BAG3.

The monitored effect of BAG3 on cell survival could be explained by its role in autophagy but might also be due to its anti-apoptotic functions. The latter are thought to be regulated by a BAG3 interaction with the anti-apoptotic factor BCL2 resulting in a protection of cells from apoptotic cell death,^53^ whereas BAG3 was also found in mitigating proteotoxicity with the consequence of an inducible resistance in tumor cells.^54, 55^ In addition, tumor cell apoptosis is reported to be induced by BAG3 silencing and improves drug-induced apoptosis of neoplastic cells.^56, 57^ Furthermore, BAG3 was described to affect the heat-shock protein 70 (HSP70)-regulated pathways intensively, which are also related to cancer cell survival and apoptosis.^58^

In line with our recent findings, the results of a proteomic analysis revealed a role for BAG3 and Major Vault Protein as potent pro-survival factors that contribute to chemotherapy resistance in breast cancer cells.^59^

As indicated in our study, immunofluorescence analysis of breast cancer patient tissue also showed that markers for autophagy were enhanced in ER*α*-positive samples. BAG3 was recently shown to be involved in cancer-signaling networks, to mediate a selective macroautophagy pathway upon protein aggregation and to attenuate proteotoxicity in cancer cells.^2, 17, 47^ Therefore, BAG3 also represents an interesting target for therapy in breast cancers that might have already acquired resistance to anti-hormone therapy. Interestingly, in our immunofluorescence analysis one sample of ER*α*-positive tissue showed a reduced signal of BAG3. But as noted above there is great variability in breast cancer and its subtypes. Therefore, the different biological behavior of these variable cancers calls for an individual and personalized medication and treatment concepts for breast cancer patients in respect to their individual disease status. For example, some clinical approaches to lower autophagic activity in cancer cells target the mTOR and PI3K complexes. Several mTOR and PI3K inhibitors or mTOR/PI3K dual inhibitors are in early phase clinical studies or already approved for therapy.^60, 61^ But for a subset of breast cancer patients that would show an ER*α*-mediated ‘*autophagy-footprint*' that is comparable to our results, these drugs would not fulfill their autophagy reducing action. This novel ER*α*-mediated non-canonical autophagy pathway, which we describe here, is mTOR and PI3K independent as the PI3K inhibitor Wortmannin did not reduce the autophagic flux in ER*α*-expressing cells and phosphorylation of mTOR is not altered. An unmet need exists for effective treatment strategies for anti-hormone resistant ER*α* breast cancer and BAG3 might represent an interesting alternative therapeutic target.

There are limited data on biomarkers and target proteins that predict the efficacy of autophagy inhibitors in breast cancer in general.^62^ Therefore, an effective dissection of the autophagy pathway is needed. Establishing autophagy networks and individual ‘*autophagy-footprints*' for patients could potentially improve effectiveness of drug treatment targeting the autophagy pathway as specialized medication for mTOR/PI3K-dependent canonical *versus* non-canonical autophagy could be applied. This presents a major challenge for personalized medicine and biomarker development but also a novel therapeutic chance for cancer patients.

## Materials and Methods

### Cell culture

Human SK-N-MC cells (ATCC HTB-10) and MCF-7 cells were obtained from the American Type Cell Collection and were cultured as described previously.^21^ All reagents for treatments were dissolved in dimethylsulfoxide (DMSO) and used in following concentrations and time intervals: 10 nM 17*β*-estradiol (E2, Sigma-Aldrich, Seelze, Germany) for 24 h; 1 *μ*M ICI 182780 (Tocris Biosciences, Bristol, UK) for 24 h; 500 nM Bafilomycin A_1_ (Enzo Life Science, Farmingdale, NY, USA) for 6 h; simultaneous treatment of 1 *μ*M Wortmannin (Sigma-Aldrich) and 100 nM Bafilomycin A_1_ for 20 h and 50 *μ*M U0126 (Promega, Madison, WI, USA) for 48 h. In case of double treatments 1 h pre-treatment with the inhibiting reagent was performed.

### Transfections

For calcium phosphate method, cells were plated in 6-well plates 24 h before transfection. All reagents were set at room temperature (RT) and 10 *μ*g plasmid DNA or 20 *μ*g siRNA was mixed with 105 *μ*l H_2_O and 15 *μ*l CaCl_2_ and incubated for 5 min. In all, 120 *μ*l 2x HEPES buffer saline was added and incubated for 30 min. The suspension was directly transferred to the medium and after 24 h fresh medium was added. For visualizing autophagic activity, GFP-RFP-LC3 plasmid (ptfLC3, Addgene, Cambridge, MA, USA) was used. After additional 24 h cells were harvested or subjected to immunocytochemistry. Generation of pIRES-ER*α* and pIRES-ER*β* was described previously^29, 30^ and pIRES plasmid was purchased from Clonetech Laboratories (Mountain View, CA, USA).

For overexpression and siRNA mediated knockdown, cells were transfected using the calcium phosphate method or FuGENE (Promega). Details are shown in the Supplementary information. Non-sense siRNA (5'-AUUCUCCGAACGUGUCACG-3') was purchased as siMAX from MWG. siRNA against BAG3 was supplied from Sigma-Aldrich (SASI_Hs02_00337266). siRNA mix against DRAM1 was supplied by MWG (No 1: 5′-ACACCUCCAGAGAGUGGUA-3′ No 2: 5′-GGAUUAUGUAUAUCACGUA-3′).

### Western blot analysis

Western blot analysis was carried out as described previously.^2^ Analysis was performed with the Fusion-SL 3500 WL system (Peqlab, Erlangen, Germany) and Aida Image Analyzer v.4v26 software (Raytest, Straubenhardt, Germany).

### Immunocytochemistry

Immunocytochemistry of cells was carried out as described previously.^2^ The paraffin-embedded tumor samples were de-paraffinized and afterwards rehydrated with in Xylol, followed by a decreasing alcohol series (100% to 70% alcohol). The rehydration was completed by incubation with bidest H_2_O and slices were stored in water until antigen detection. Afterwards immunostaining was performed as described previously.^2^ Cells and tissue were analyzed by microscopy using an inverted Leica TCS SP5 (Wetzlar, Germany) and Zeiss LSM710 meta confocal microscope (Oberkochen, Germany) and images were processed with Adobe Photoshop CS5 (San Jose, CA, USA) and Leica LAS AF lite software (Leica Microsystems (UK) Ltd, Milton Keynes, UK). GFP-RFP-LC3 dot formation (visualized using the GFP-RFP-LC3 expression plasmid mentioned above) was quantified by counting dots in confocal laser scanning microscopic pictures of corresponding transfected cell lines and after Bafilomycin A_1_ treatment (500 nM, 6 h). At least 56 cells per cell line were analyzed.

### Transmission electron microscopy

Specimen preparation and transmission electron microscopy analysis were carried out as described previously^63^ and images were processed with Adobe Photoshop CS5.

### Antibodies

The antibodies used for immunocytochemistry as well as for western blot analysis in this study were as follows: ATG7 (8558, Cell Signaling Technologies, Danvers, MA, USA); BAG3 (ab47124, Abcam, Cambridge, UK); BCL2 (sc-492, Santa Cruz, Dallas, TX, USA); ER*α* (RM9101S0, Thermo Fischer Scientific, Waltham, MA, USA); ERK1/2 (9102, Cell Signaling Technologies); p-ERK1/2 (9106, Cell Signaling Technologies); LC3B (L7543, Sigma-Aldrich); mTOR (OP97, Millipore, Billerica, MA, USA); p-mTOR S2448 (ab51044, Abcam); NBR1 (00004077-M01, Abnova, Taipei, Taiwan); SQSTM1 (GP62-C, Progen, Heidelberg, Germany); PI3K Class III (4263, Cell Signaling Technologies); Tubulin (T9026, Sigma-Aldrich); WIPI1 (HPA007493, Sigma-Aldrich); Beclin1 (ab51031; Abcam); p70S6K (9202, Cell Signaling); p70S6K Thr389 (9206, Cell Signaling). Secondary antibodies were anti-mouse/rabbit/guinea pig antibodies conjugated to DyLight 488, 649 and Cy3 (immunofluorescence, Jackson ImmunoResearch, West Grove, PA, USA) or to HRP (immunoblotting, Jackson ImmunoResearch).

### Human breast cancer tissue

Human breast cancer tumor samples were acquired by surgery from cancer patients. Details about cancer patients can be found in Supplementary Table 1. Informed consent was obtained from all subjects and studies were approved by the Institutional Review Boards and Ethical Committee of the collaborating Centrum für Tumorerkrankungen (UCT) of the University of Frankfurt.

### PCR, reverse transcription PCR and quantitative real-time PCR

PCR and quantitative real-time PCR were carried out as described previously^21^ using HATPL-1 (Biomol, Hamburg, Germany) according to the manufacturer's protocol. Fold changes and *P*-values of target genes in SK-ER*α*, SK-ER*β* and MCF-7 cells compared with SK-01 cells were calculated using RT^2^ Profiler PCR Array Data Analysis Template v4.0 from Qiagen (Venlo, Netherlands). All eight housekeeping genes spotted on the array plate were used as internal controls. Only genes with a higher fold change than +1.5 or −1.5 and a *P*-value of *P*≤0.05 were treated as regulated genes.

For the detection of DRAM1 siRNA knock down, we used primer supplied by Qiagen (Hs_DRAM1_1_SG). The housekeeping gene RPL19 was detected with the following primer sequences: forward 5'-GAAATCGCCAATGCCAACTC-3' and reverse 5'-TTCCTTGGTCTTAGACCTGCG-3'.

Primer sequences used for characterizing ER*α* and ER*β*-positive and -negative cell lines are as follows: ER*α* forward 5′-GTGCCAGGCTTTGTGGATTTG-3′ ER*α* reverse 5′-GTTACTCATGTGCCTGATGTG-3′ ER*β* forward 5′-GAGGCCTCCATGATGATGTC-3′ ER*β* reverse 5′-TCTCCAGCAGCAGGTCAT-3′ and pIRES-ER*α* and pIRES-ER*β* were used as positive controls. Samples were analyzed on a 1% agarose (Biozym, Landgraaf, Netherlands) gel. Mass Ruler DNA ladder (#SM0403, Fermentas, Thermo Scientific, Waltham, MA, USA) was used as a reference.

### ERE-luciferase assay

SK-01, SK-ER*α*, SK-ER*β* and MCF-7 cells were transfected with the ERE-Luc reporter plasmid containing EREs fused to the firefly luciferase gene (D-MTV-ERE-LUC;^64^). Transfection was carried out as described above. After 24 h cells were stimulated with 17*β*-estradiol (10 nM) and/or ICI (1 *μ*M) for 24 h. Subsequently, cells were harvested by using the Luciferase Assay System (Promega, Cat. No. E4030). The protein concentrations were determined by BCA as described above and the luminescence readings were assigned in an automatic counter (Wallac Victor2, PerkinElmer, Waltham, MA, USA). Transfection experiments were performed in triplicates, repeated three times and normalized for identical protein contend.

### Survival experiments by fluorescence activated cell sorting (FACS)

Twenty-four hours before stimulation SK-01, SK-ER*α*, SK-ER*β* and MCF-7 cells were seeded in 24-well plates. Following 1 h pre-incubation with Bafilomycin A_1_ (500 nM) or DMSO, cells were treated with vehicle control, 400 *μ*M or 600 *μ*M H_2_O_2_ (Sigma-Aldrich) for 24 h. After treatment, the supernatant from the cells was collected and cells were dissolved from the plates with Trypsin digestion and again incubated with the prior collected supernatant. Following 5 min of centrifugation (800* g*) the cells were resolved in 100 *μ*l PBS. All steps were carried out on ice. Cell viability was measured by staining cells with propidium iodide (PI) (1 *μ*g/ml). PI fluorescence was determined with a FACScan Flow Cytometer (Becton Dickinson, Franklin Lakes, NJ, USA). Cell populations were pre-gated by forward scatter (FSC-Height) and side scatter (SSC-Height). Afterwards, results were analyzed with the BD CellQuest Pro Analysis software.

### Statistical analysis

Quantitative data are expressed as the means±S.E.M. Statistical comparisons between experimental groups were made using Student's *t-*test. Probability values of *P*≤0.05 were considered as significant.

## Abbreviations

AE: anti-estrogens
Atg: autophagy-related
BafA_1_: Bafilomycin A_1_
BAG3: Bcl2-associated athanogene 3
BCA: bicinchoninic acid
Bcl2: B-cell CLL/lymphoma 2
DABCO: 1,4-Diaza-bicyclo(2,2,2)octan
DAPI: 4,6-diamidino-2-phenylindole
DMSO: dimethylsulfoxide
DRAM1: DNA-damage regulated autophagy modulator 1
E2: 17*β*-estradiol
ER: estrogen receptor
ERE: estrogen response element
ERK: extracellular signal-regulated protein kinase
FACS: fluorescence-activated cell sorting
FIP200: Fak family kinase-interacting protein 200kDa
GABARAP: GABA receptor associated protein
GFP: green fluorescent protein
HER2: human epidermal growth factor receptor 2
HRP: horseradish peroxidase
HTPL-1: human autophagy primer library 1
ICI: ICI 182780
MAP1LC3/LC3: microtubule-associated protein 1 light chain 3
MAPK: Mitogen-activated protein kinase
mTOR: mammalian target of rapamycin
mTORC1: mammalian target of rapamycin complex 1
NBR1: Neighbor of Brca 1
PI: propidium iodide
PI3K: phosphatidylinositol 3 kinase
PI3KCIII: phosphatidylinositol 3 kinase class III
RFP: red fluorescent protein
SQSTM1: Sequestosome 1
ULK1: UNC-51-like kinase 1
WIPI: WD repeat domain phosphoinositide interacting
WN: Wortmannin

## References

[bib1] 1Choi AM, Ryter SW, Levine B. Autophagy in human health and disease. N Engl J Med 2013; 368: 651–662. 23406030 10.1056/NEJMra1205406

[bib2] 2Gamerdinger M, Hajieva P, Kaya AM, Wolfrum U, Hartl FU, Behl C. Protein quality control during aging involves recruitment of the macroautophagy pathway by BAG3. EMBO J 2009; 28: 889–901. 19229298 10.1038/emboj.2009.29 PMC2647772

[bib3] 3Mizushima N, Levine B, Cuervo AM, Klionsky DJ. Autophagy fights disease through cellular self-digestion. Nature 2008; 451: 1069–1075. 18305538 10.1038/nature06639 PMC2670399

[bib4] 4Ravikumar B, Sarkar S, Davies JE, Futter M, Garcia-Arencibia M, Green-Thompson ZW et al. Regulation of mammalian autophagy in physiology and pathophysiology. Physiol Rev 2010; 90: 1383–1435. 20959619 10.1152/physrev.00030.2009

[bib5] 5Boya P, Reggiori F, Codogno P. Emerging regulation and functions of autophagy. Nat Cell Biol 2013; 15: 713–720. 23817233 10.1038/ncb2788 PMC7097732

[bib6] 6Mizushima N, Komatsu M. Autophagy: renovation of cells and tissues. Cell 2011; 147: 728–741. 22078875 10.1016/j.cell.2011.10.026

[bib7] 7Rubinsztein DC, Marino G, Kroemer G. Autophagy and aging. Cell 2011; 146: 682–695. 21884931 10.1016/j.cell.2011.07.030

[bib8] 8Codogno P, Mehrpour M, Proikas-Cezanne T. Canonical and non-canonical autophagy: variations on a common theme of self-eating? Nat Rev Mol Cell Biol 2012; 13: 7–12. 10.1038/nrm3249 22166994

[bib9] 9Scarlatti F, Maffei R, Beau I, Codogno P, Ghidoni R. Role of non-canonical Beclin 1-independent autophagy in cell death induced by resveratrol in human breast cancer cells. Cell Death Differ 2008; 15: 1318–1329. 18421301 10.1038/cdd.2008.51

[bib10] 10Cheong H, Lindsten T, Wu J, Lu C, Thompson CB. Ammonia-induced autophagy is independent of ULK1/ULK2 kinases. Proc Natl Acad Sci USA 2011; 108: 11121–11126. 21690395 10.1073/pnas.1107969108 PMC3131371

[bib11] 11Sarkar S, Floto RA, Berger Z, Imarisio S, Cordenier A, Pasco M et al. Lithium induces autophagy by inhibiting inositol monophosphatase. J Cell Biol 2005; 170: 1101–1111. 16186256 10.1083/jcb.200504035 PMC2171537

[bib12] 12Hiebel C, Kromm T, Stark M, Behl C. Cannabinoid receptor 1 modulates the autophagic flux independent of mTOR- and BECLIN1-complex. J Neurochem 2014; 131: 484–497. 25066892 10.1111/jnc.12839

[bib13] 13Degenhardt K, Mathew R, Beaudoin B, Bray K, Anderson D, Chen G et al. Autophagy promotes tumor cell survival and restricts necrosis, inflammation, and tumorigenesis. Cancer Cell 2006; 10: 51–64. 16843265 10.1016/j.ccr.2006.06.001 PMC2857533

[bib14] 14Cheong H, Lu C, Lindsten T, Thompson CB. Therapeutic targets in cancer cell metabolism and autophagy. Nat Biotechnol 2012; 30: 671–678. 22781696 10.1038/nbt.2285 PMC3876738

[bib15] 15Berardi DE, Campodonico PB, Diaz Bessone MI, Urtreger AJ, Todaro LB. Autophagy: friend or foe in breast cancer development, progression, and treatment. Int J Breast Cancer 2011; 2011: 595092. 22295229 10.4061/2011/595092 PMC3262577

[bib16] 16Apel A, Herr I, Schwarz H, Rodemann HP, Mayer A. Blocked autophagy sensitizes resistant carcinoma cells to radiation therapy. Cancer Res 2008; 68: 1485–1494. 18316613 10.1158/0008-5472.CAN-07-0562

[bib17] 17Rapino F, Jung M, Fulda S. BAG3 induction is required to mitigate proteotoxicity via selective autophagy following inhibition of constitutive protein degradation pathways. Oncogene 2013; 33: 1713–1724. 23644654 10.1038/onc.2013.110

[bib18] 18Galavotti S, Bartesaghi S, Faccenda D, Shaked-Rabi M, Sanzone S, McEvoy A et al. The autophagy-associated factors DRAM1 and p62 regulate cell migration and invasion in glioblastoma stem cells. Oncogene 2013; 32: 699–712. 22525272 10.1038/onc.2012.111

[bib19] 19Jo YK, Kim SC, Park IJ, Park SJ, Jin DH, Hong SW et al. Increased expression of ATG10 in colorectal cancer is associated with lymphovascular invasion and lymph node metastasis. PLoS One 2012; 7: e52705. 23285162 10.1371/journal.pone.0052705 PMC3527592

[bib20] 20Lorin S, Hamai A, Mehrpour M, Codogno P. Autophagy regulation and its role in cancer. Semin Cancer Biol 2013; 23: 361–379. 23811268 10.1016/j.semcancer.2013.06.007

[bib21] 21Brendel A, Felzen V, Morawe T, Manthey D, Behl C. Differential regulation of apoptosis-associated genes by estrogen receptor alpha in human neuroblastoma cells. Restor Neurol Neurosci 2013; 31: 199–211. 23271418 10.3233/RNN-120272

[bib22] 22Cleator S, Heller W, Coombes RC. Triple-negative breast cancer: therapeutic options. Lancet Oncol 2007; 8: 235–244. 17329194 10.1016/S1470-2045(07)70074-8

[bib23] 23Konecny G, Pauletti G, Pegram M, Untch M, Dandekar S, Aguilar Z et al. Quantitative association between HER-2/neu and steroid hormone receptors in hormone receptor-positive primary breast cancer. J Natl Cancer Inst 2003; 95: 142–153. 12529347 10.1093/jnci/95.2.142

[bib24] 24Dawson SJ, Provenzano E, Caldas C. Triple negative breast cancers: clinical and prognostic implications. Eur J Cancer 2009; 45: 27–40. 19775602 10.1016/S0959-8049(09)70013-9

[bib25] 25Bieche I, Parfait B, Laurendeau I, Girault I, Vidaud M, Lidereau R. Quantification of estrogen receptor alpha and beta expression in sporadic breast cancer. Oncogene 2001; 20: 8109–8115. 11781824 10.1038/sj.onc.1204917

[bib26] 26Ma XJ, Wang Z, Ryan PD, Isakoff SJ, Barmettler A, Fuller A et al. A two-gene expression ratio predicts clinical outcome in breast cancer patients treated with tamoxifen. Cancer Cell 2004; 5: 607–616. 15193263 10.1016/j.ccr.2004.05.015

[bib27] 27True O, Matthias P. Interplay between histone deacetylases and autophagy—from cancer therapy to neurodegeneration. Immunol Cell Biol 2012; 90: 78–84. 22124372 10.1038/icb.2011.103

[bib28] 28Manthey D, Behl C. From structural biochemistry to expression profiling: neuroprotective activities of estrogen. Neuroscience 2006; 138: 845–850. 16343783 10.1016/j.neuroscience.2005.10.058

[bib29] 29Manthey D, Heck S, Engert S, Behl C. Estrogen induces a rapid secretion of amyloid beta precursor protein via the mitogen-activated protein kinase pathway. Eur J Biochem 2001; 268: 4285–4291. 11488923 10.1046/j.1432-1327.2001.02346.x

[bib30] 30Zschocke J, Manthey D, Bayatti N, van der Burg B, Goodenough S, Behl C. Estrogen receptor alpha-mediated silencing of caveolin gene expression in neuronal cells. J Biol Chem 2002; 277: 38772–38780. 12138116 10.1074/jbc.M205664200

[bib31] 31Gamerdinger M, Manthey D, Behl C. Oestrogen receptor subtype-specific repression of calpain expression and calpain enzymatic activity in neuronal cells—implications for neuroprotection against Ca-mediated excitotoxicity. J Neurochem 2006; 97: 57–68. 16524385 10.1111/j.1471-4159.2006.03675.x

[bib32] 32Liu BQ, Du ZX, Zong ZH, Li C, Li N, Zhang Q et al. BAG3-dependent noncanonical autophagy induced by proteasome inhibition in HepG2 cells. Autophagy 2013; 9: 905–916. 23575457 10.4161/auto.24292 PMC3672299

[bib33] 33Manthey D, Gamerdinger M, Behl C. The selective beta1-adrenoceptor antagonist nebivolol is a potential oestrogen receptor agonist with neuroprotective abilities. Br J Pharmacol 2010; 159: 1264–1273. 20128815 10.1111/j.1476-5381.2009.00610.x PMC2848930

[bib34] 34Zschocke J, Manthey D, Bayatti N, Behl C. Functional interaction of estrogen receptor alpha and caveolin isoforms in neuronal SK-N-MC cells. J Steroid Biochem Mol Biol 2003; 84: 167–170. 12711000 10.1016/s0960-0760(03)00026-8

[bib35] 35Gattelli A, Nalvarte I, Boulay A, Roloff TC, Schreiber M, Carragher N et al. Ret inhibition decreases growth and metastatic potential of estrogen receptor positive breast cancer cells. EMBO Mol Med 2013; 5: 1335–1350. 23868506 10.1002/emmm.201302625 PMC3799490

[bib36] 36Johansen T, Lamark T. Selective autophagy mediated by autophagic adapter proteins. Autophagy 2011; 7: 279–296. 21189453 10.4161/auto.7.3.14487 PMC3060413

[bib37] 37Chiang GG, Abraham RT. Phosphorylation of mammalian target of rapamycin (mTOR) at Ser-2448 is mediated by p70S6 kinase. J Biol Chem 2005; 280: 25485–25490. 15899889 10.1074/jbc.M501707200

[bib38] 38Meijer AJ, Codogno P. Regulation and role of autophagy in mammalian cells. Int J Biochem Cell Biol 2004; 36: 2445–2462. 15325584 10.1016/j.biocel.2004.02.002

[bib39] 39Kang R, Zeh HJ, Lotze MT, Tang D. The Beclin 1 network regulates autophagy and apoptosis. Cell Death Differ 2011; 18: 571–580. 21311563 10.1038/cdd.2010.191 PMC3131912

[bib40] 40Galluzzi L, Pietrocola F, Bravo-San Pedro JM, Amaravadi RK, Baehrecke EH, Cecconi F et al. Autophagy in malignant transformation and cancer progression. Embo J 2015; 34: 856–880. 25712477 10.15252/embj.201490784 PMC4388596

[bib41] 41Gaugel A, Bakula D, Hoffmann A, Proikas-Cezanne T. Defining regulatory and phosphoinositide-binding sites in the human WIPI-1 beta-propeller responsible for autophagosomal membrane localization downstream of mTORC1 inhibition. J Mol Signal 2012; 7: 16. 23088497 10.1186/1750-2187-7-16 PMC3543385

[bib42] 42Kimura S, Noda T, Yoshimori T. Dissection of the autophagosome maturation process by a novel reporter protein, tandem fluorescent-tagged LC3. Autophagy 2007; 3: 452–460. 17534139 10.4161/auto.4451

[bib43] 43Klionsky DJ, Abdalla FC, Abeliovich H, Abraham RT, Acevedo-Arozena A, Adeli K et al. Guidelines for the use and interpretation of assays for monitoring autophagy. Autophagy 2012; 8: 445–544. 22966490 10.4161/auto.19496 PMC3404883

[bib44] 44Gorrini C, Harris IS, Mak TW. Modulation of oxidative stress as an anticancer strategy. Nat Rev Drug Discov 2013; 12: 931–947. 24287781 10.1038/nrd4002

[bib45] 45Schoenlein PV, Periyasamy-Thandavan S, Samaddar JS, Jackson WH, Barrett JT. Autophagy facilitates the progression of ERalpha-positive breast cancer cells to antiestrogen resistance. Autophagy 2009; 5: 400–403. 19221464 10.4161/auto.5.3.7784

[bib46] 46Gonzalez Y, Aryal B, Chehab L, Rao VA. Atg7- and Keap1-dependent autophagy protects breast cancer cell lines against mitoquinone-induced oxidative stress. Oncotarget 2014; 5: 1526–1537. 24681637 10.18632/oncotarget.1715 PMC4039229

[bib47] 47Colvin TA, Gabai VL, Gong J, Calderwood SK, Li H, Gummuluru S et al. Hsp70-Bag3 interactions regulate cancer-related signaling networks. Cancer Res 2014; 74: 4731–4740. 24994713 10.1158/0008-5472.CAN-14-0747 PMC4174322

[bib48] 48Cook KL, Shajahan AN, Clarke R. Autophagy and endocrine resistance in breast cancer. Exp Rev Anticancer Ther 2011; 11: 1283–1294. 10.1586/era.11.111 PMC3701722 21916582

[bib49] 49Behl C. Oestrogen as a neuroprotective hormone. Nat Rev Neurosci 2002; 3: 433–442. 12042878 10.1038/nrn846

[bib50] 50Simoncini T, Hafezi-Moghadam A, Brazil DP, Ley K, Chin WW, Liao JK. Interaction of oestrogen receptor with the regulatory subunit of phosphatidylinositol-3-OH kinase. Nature 2000; 407: 538–541. 11029009 10.1038/35035131 PMC2670482

[bib51] 51Mauthe M, Jacob A, Freiberger S, Hentschel K, Stierhof YD, Codogno P et al. Resveratrol-mediated autophagy requires WIPI-1-regulated LC3 lipidation in the absence of induced phagophore formation. Autophagy 2011; 7: 1448–1461. 22082875 10.4161/auto.7.12.17802 PMC3288019

[bib52] 52Gamerdinger M, Kaya AM, Wolfrum U, Clement AM, Behl C. BAG3 mediates chaperone-based aggresome-targeting and selective autophagy of misfolded proteins. EMBO Rep 2011; 12: 149–156. 21252941 10.1038/embor.2010.203 PMC3049430

[bib53] 53Knezevic T, Myers VD, Gordon J, Tilley DG, Sharp TE 3rd, Wang J et al. BAG3: a new player in the heart failure paradigm. Heart Fail Rev 2015; 20: 423–434. 25925243 10.1007/s10741-015-9487-6 PMC4463985

[bib54] 54Rapino F, Jung M, Fulda S. BAG3 induction is required to mitigate proteotoxicity via selective autophagy following inhibition of constitutive protein degradation pathways. Oncogene 2014; 33: 1713–1724. 23644654 10.1038/onc.2013.110

[bib55] 55Rapino F, Abhari BA, Jung M, Fulda S. NIK is required for NF-kappaB-mediated induction of BAG3 upon inhibition of constitutive protein degradation pathways. Cell Death Dis 2015; 6: e1692. 25766331 10.1038/cddis.2014.584 PMC4385908

[bib56] 56Rosati A, Graziano V, De Laurenzi V, Pascale M, Turco MC. BAG3: a multifaceted protein that regulates major cell pathways. Cell Death Dis 2011; 2: e141. 21472004 10.1038/cddis.2011.24 PMC3122056

[bib57] 57Cotugno R, Basile A, Romano E, Gallotta D, Belisario MA. BAG3 down-modulation sensitizes HPV18(+) HeLa cells to PEITC-induced apoptosis and restores p53. Cancer Lett 2014; 354: 263–271. 25175321 10.1016/j.canlet.2014.08.022 PMC7116956

[bib58] 58Sherman MY, Gabai VL. Hsp70 in cancer: back to the future. Oncogene 2014. 10.1038/onc.2014.349 PMC4411196 25347739

[bib59] 59Pasillas MP, Shields S, Reilly R, Strnadel J, Behl C, Park R et al. Proteomic analysis reveals a role for Bcl2-associated athanogene 3 and Major Vault Protein in resistance to apoptosis in senescent cells by regulating ERK1/2 activation. Mol Cell Proteomics 2014; 14: 1–14. 24997994 10.1074/mcp.M114.037697 PMC4288246

[bib60] 60Davis NM, Sokolosky M, Stadelman K, Abrams SL, Libra M, Candido S et al. Deregulation of the EGFR/PI3K/PTEN/Akt/mTORC1 pathway in breast cancer: possibilities for therapeutic intervention. Oncotarget 2014; 5: 4603–4650. 25051360 10.18632/oncotarget.2209 PMC4148087

[bib61] 61Vicier C, Dieci M, Arnedos M, Delaloge S, Viens P, Andre F. Clinical development of mTOR inhibitors in breast cancer. Breast Cancer Res 2014; 16: 203. 25189767 10.1186/bcr3618 PMC3978635

[bib62] 62Gonzalez-Angulo AM, Blumenschein GR Jr. Defining biomarkers to predict sensitivity to PI3K/Akt/mTOR pathway inhibitors in breast cancer. Cancer Treat Rev 2013; 39: 313–320. 23218708 10.1016/j.ctrv.2012.11.002 PMC3604032

[bib63] 63Mersseman V, Besold K, Reddehase MJ, Wolfrum U, Strand D, Plachter B et al. Exogenous introduction of an immunodominant peptide from the non-structural IE1 protein of human cytomegalovirus into the MHC class I presentation pathway by recombinant dense bodies. J Gen Virol 2008; 89: 369–379. 18198367 10.1099/vir.0.83380-0

[bib64] 64Behl C, Widmann M, Trapp T, Holsboer F. 17-beta estradiol protects neurons from oxidative stress-induced cell death *in vitro*. Biochem Biophys Res Commun 1995; 216: 473–482. 7488136 10.1006/bbrc.1995.2647

