# Supplementary material for: Estrogen receptor α regulates non-canonical autophagy that provides stress resistance to neuroblastoma and breast cancer cells and involves BAG3 function
Source: Cell Death Dis. 2015 Jul 9;6(7):e1812–. doi: 10.1038/cddis.2015.181 (PMC4650728; doi:10.1038/cddis.2015.181)
Supplement: Supplementary Information [file cddis2015181x1.doc]

**Supplementary Information:**

Estrogen receptor α regulates non-canonical autophagy that provides stress resistance to neuroblastoma and breast cancer cells and involves BAG3 function

Running title: ERα-regulated non-canonical autophagy

**Vanessa Felzen^1^, Christof Hiebel^1^, Ingrid Koziollek-Drechsler^1^, , Sonja Reißig^2^, Uwe Wolfrum^3^, Donat Kögel^4^, Christian Brandts^5^, Christian Behl^1,*^, and Tobias Morawe^1,*^**

*^1^Institute of Pathobiochemistry, University Medical Center of the Johannes Gutenberg University, Duesbergweg 6, 55099 Mainz, Germany*

*^2^Institute of Molecular Medicine, University Medical Center of the Johannes Gutenberg University, Obere Zahlbacher Straße 67, 55131 Mainz, Germany*

*^3^Department of Cell and Matrix Biology, Institute of Zoology, Johannes Gutenberg University Mainz, Muellerweg 6, 55099 Mainz, Germany*

*^4^Experimental Neurosurgery, Neuroscience Center, Goethe University Hospital, Theodor-Stern-Kai 7, 60590 Frankfurt, Germany*

*^5^Department of Medicine, Hematology/Oncology and University Cancer Center Frankfurt, Goethe University Hospital, Theodor-Stern-Kai 7, 60595 Frankfurt, Germany*

*** Authors for correspondence:**

Institute of Pathobiochemistry

University Medical Center of the Johannes Gutenberg University

Duesbergweg 6, 55099 Mainz, Germany

Tel: ++ 49 (0) 6131 39 25890

Fax: ++ 49 (0) 6131 39 25792

E-mail: morawe@uni-mainz.de; cbehl@uni-mainz.de

**Supplementary Table 1:** **Patient data of breast cancer tissue from individuals with diagnosed Estrogen receptor status used for immunofluorescence analysis.**

**Supplementary Figure 1: Characterization of the different ER negative and ERα and ERβ expressing cells.** (A) PCR analysis with specific primer pairs directed against ERα and ERβ reveals that only SK-ERα and MCF-7 cells express ERα and exclusively SK-ERβ express ERβ whereas SK-ERα and MCF-7 cells are ERβ negative. Mock transfected SK-01 controls do not express any ER. pIRES-ERα and pIRES-ERβ plasmids initially used for stable transfection were used as positive controls and PCR sample containing dH_2_O instead of DNA as negative control (mock). (B) Two stable subclones of each transfectant, mock (SK-01 and SK-02), ERα (SK-ERα4 and SK-ERα9) and ERβ (SK-ERβ8 and SK-ERβ38) showed the same autophagic activity judged via LC3-II accumulation as the corresponding counterpart. Autophagic flux was determined by the accumulation of LC3-II in a 6 h treatment period with 500 nM BafA_1_.

**Supplementary Figure 2: ERα expression does not alter the ratio of p70S6K to phosphor p70S6K.** Western blot analysis of protein extracts from untreated cells were performed for detection of p70S6K and phosphorylated p70S6K. In the diagram the ratio between phosphorylated and unphosphorylated protein after normalization to Tubulin is depicted. Values of 3 independent experiments are expressed as mean ± s.e.m. and control SK-01 cells were set to 100%. No statistical significant difference was observed.

**Supplementary Figure 3:** **Beclin1 knockdown enhances the autophagic flux independent of ERα expression.** SK-01, SK-ERα and MCF-7 cells were transfected with nonsense siRNA (siNS) and Beclin1 siRNA (siBeclin1) for 48 h, as indicated. Protein extracts from vehicle or BafA_1_ treated cells were subjected to Western blot analysis and calculation of autophagic flux using anti-LC3 antibody and knockdown efficiency was determined by using anti-Beclin1 antibody. Tubulin was used as loading control. Autophagic flux was determined by the accumulation of LC3-II in a 6 h treatment period with 500 nM BafA_1_. Therefore, normalized LC3-II levels in the absence of the lysosomal inhibitor were subtracted from corresponding levels obtained in the presence of BafA_1_. Values of 3 independent experiments in each panel are expressed as mean ± s.e.m. (*) on bars represent statistical significance of p<0.05 comparing 2 groups and n.s. displays no statistical significant difference.

**Supplementary Figure 4: Inhibiting canonical autophagy via Wortmannin and the MAPK pathway with U0126 does not reduce the autophagic flux in ERα positive cells.** (A) SK-01, SK-ERα and MCF-7 cells were treated with vehicle control, Wortmannin (1 µM) and BafA_1_ (100 nM) for 20 h and were subjected to Western blot analysis. Autophagic flux was determined by the accumulation of LC3-II and SQSTM1. Tubulin was used as loading control. (B) SK-01, SK-ERα, SK-ERβ and MCF-7 cells were treated with vehicle control, 50 µM U0126 for 48 h and 50 µM U0126 for 48 h plus 500 nM BafA_1_ for the last 6 h of U0126 treatment and subjected to western blot analysis. U0126 efficacy was determined by phosphorylated ERK1/2 level compared to total ERK1/2 protein levels. Autophagic flux was determined by the accumulation of LC3-II and Tubulin was used as loading control.

**Supplementary Figure 5: DRAM1 knockdown reduces autophagic activity in ERα-expressing cell and ERα overexpression leads to an enhanced autophagic flux and elevated BAG3 protein level in SK-N-MC cells.** (a) SK-01, SK-ERα and MCF-7 cells were transfected with nonsense siRNA (siNS) and DRAM1 siRNA (siDRAM1) for 48 h, as indicated. Protein extracts from vehicle or BafA_1_ treated cells were subjected to Western blot analysis and calculation of autophagic flux using anti-LC3 antibody and knockdown efficiency was determined by qPCR analysis. For Western blot analysis Tubulin was used as loading control. (b) MCF-7 cells were transfected with nonsense siRNA (siNS) and ERα siRNA (siERα) for 48 h, as indicated. Protein extracts from vehicle or BafA_1_ treated cells were subjected to Western blot analysis and calculation of autophagic flux using anti-LC3 antibody and knockdown efficiency was determined by using anti-ERα antibody. Tubulin was used as loading control. Overexpression of ERα in SK-N-MC cells was performed by transient transfection of pIRES-ERα plasmid. Transfection efficiency and calculation of autophagic flux was determined by using anti-ERα and anti-LC3 antibody, respectively. Tubulin was used as loading control. Autophagic flux was determined by the accumulation of LC3-II in a 6 h treatment period with 500 nM BafA_1_. Therefore, normalized LC3-II levels in the absence of the lysosomal inhibitor were subtracted from corresponding levels obtained in the presence of BafA_1_. Values of 3 independent experiments in each panel are expressed as mean ± s.e.m. (*) on bars represent statistical significance of p<0.05 comparing 2 groups and n.s. displays no statistical significant difference.

**Supplementary Figure 6: Estrogen receptor reporter assay.** SK-01, SK-ERα, SK-ERβ and MCF-7 cells were treated with vehicle control, E2 (10 nM), ICI (1 µM) or a co-treatment with E2 and ICI for 24 h. Activation of the ER response element (ERE) was plotted as Luciferase units in % of control cells (SK-01). Values of 3 independent experiments are expressed as mean ± s.e.m..

**Supplementary Figure 7:** **Estrogen receptors differentially regulate autophagy pathway-associated gene expression independent of ERE-mediated transcription factor activity.** Total RNA from SK-01, SK-ERα, SK-ERβ and MCF-7 cells was characterized by using the Human Autophagy Primer Library 1 (HATPL-1) comparing ER expressing cells to mock-plasmid transfected controls (SK-01) after vehicle control, E2 (10 nM) or ICI (1 µM) treatment for 24 h. Red numbers indicate an upregulation greater than 1.5 fold, blue numbers indicate an downregulation greater than 1.5 fold, dark red numbers denote a p-value<0.05, black numbers depict no significant change and n.s. no significant change in gene expression within the whole group. Results represent the mean values of five independent experiments. 3D-profile analysis summing up the PCR array data comparing ER expressing cells to control cells shows that there is no major change in gene expression in autophagy pathway associated genes.

**Supplementary Figure 8: Enhanced expression of autophagy makers in ERα-positive breast cancer patients.** Formalin-fixed paraffin-embedded samples from ER-positive and ER-negative breast cancer patients stained for ERα, BAG3, LC3, SQSTM1 and DAPI, were used for immunocytochemistry, as indicated. Images acquired by confocal microscopy are shown. Insets (white dotted line) show regions of interest with high ERα expression. Scale bars: 50 µm.
