# Supplementary figures and images for: Estrogen receptor α regulates non-canonical autophagy that provides stress resistance to neuroblastoma and breast cancer cells and involves BAG3 function
Source: Cell Death Dis. 2015 Jul 9;6(7):e1812–. doi: 10.1038/cddis.2015.181 (PMC4650728; doi:10.1038/cddis.2015.181)

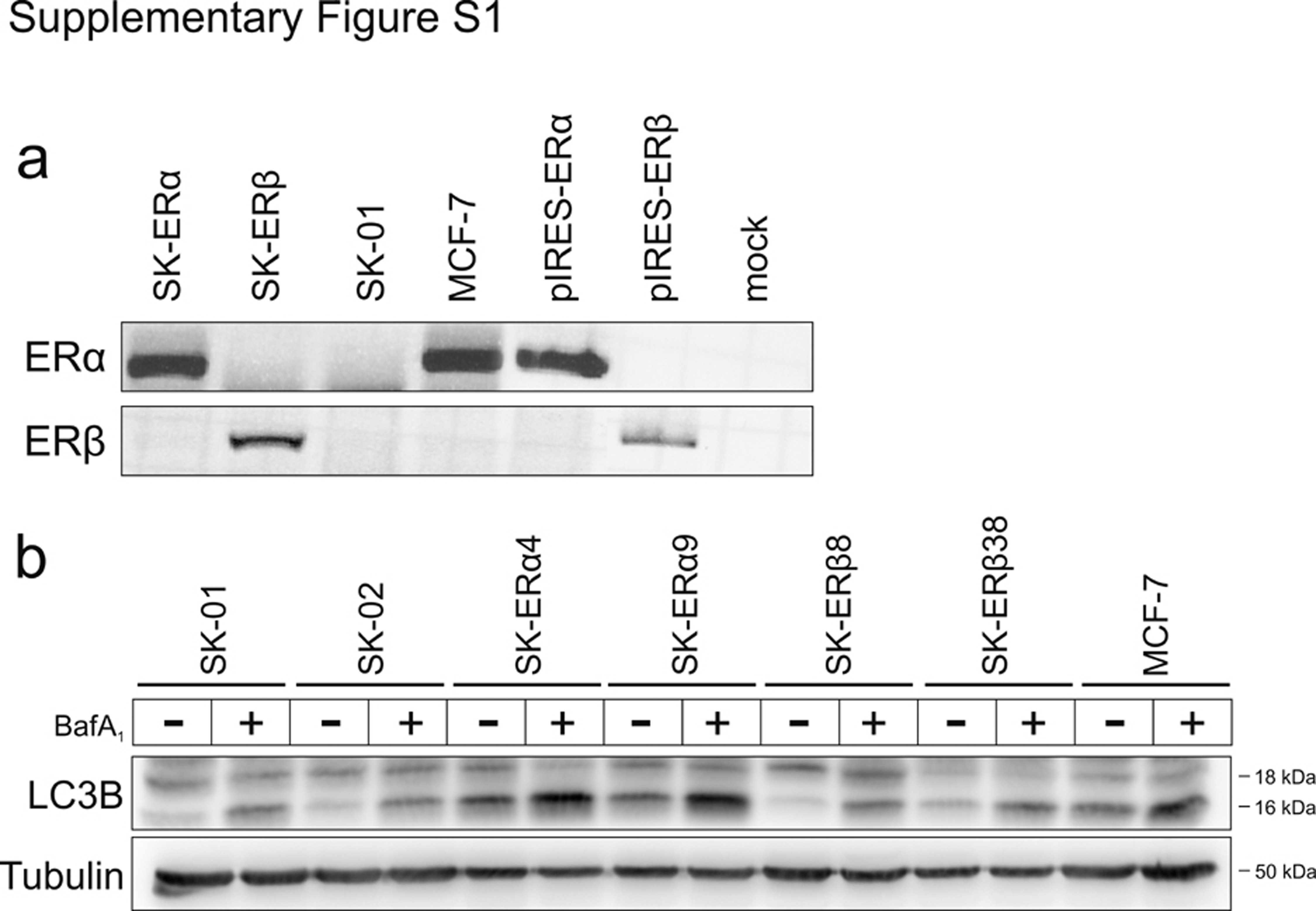

Supplement: Supplementary Figure 1 [file cddis2015181x2.tif]

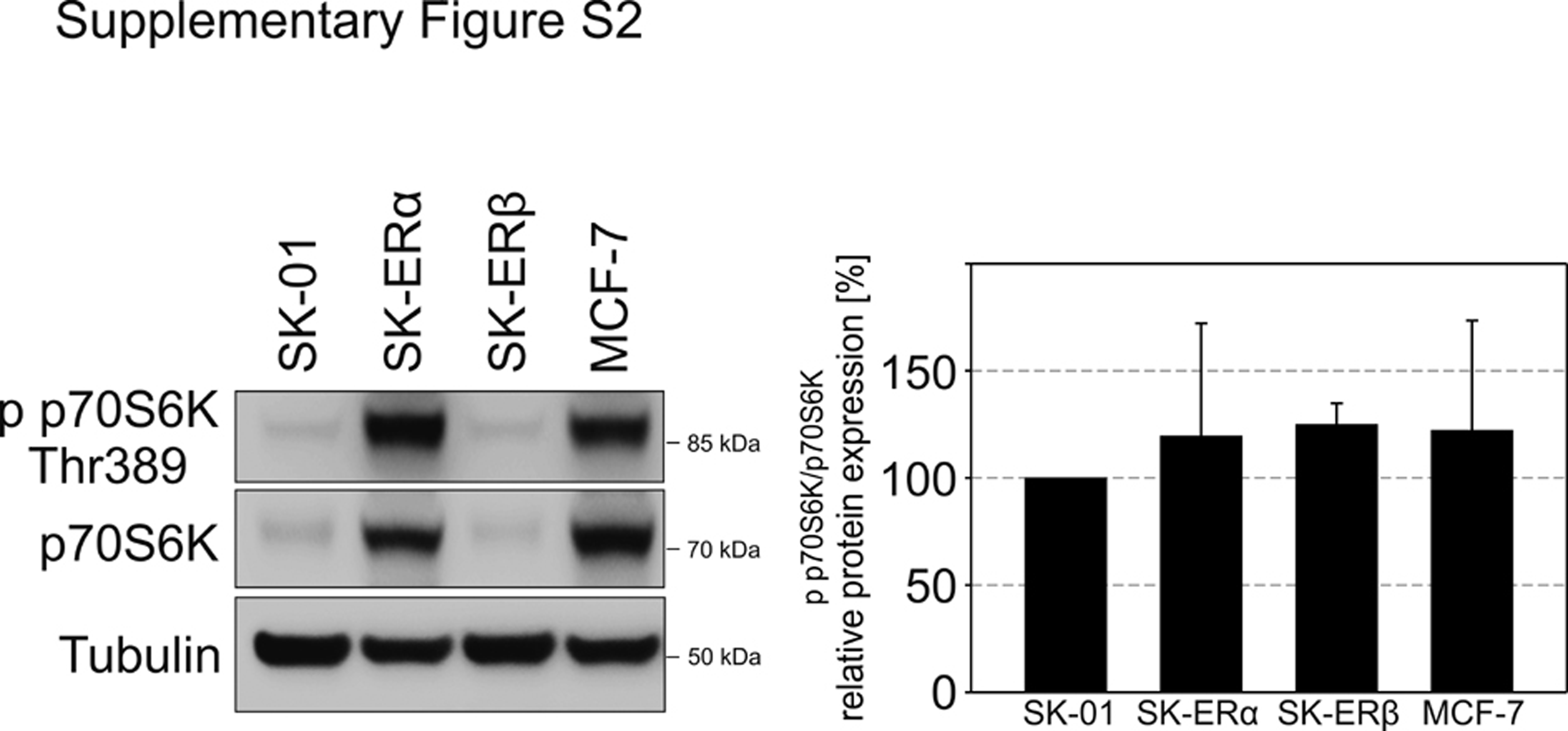

Supplement: Supplementary Figure 2 [file cddis2015181x3.tif]

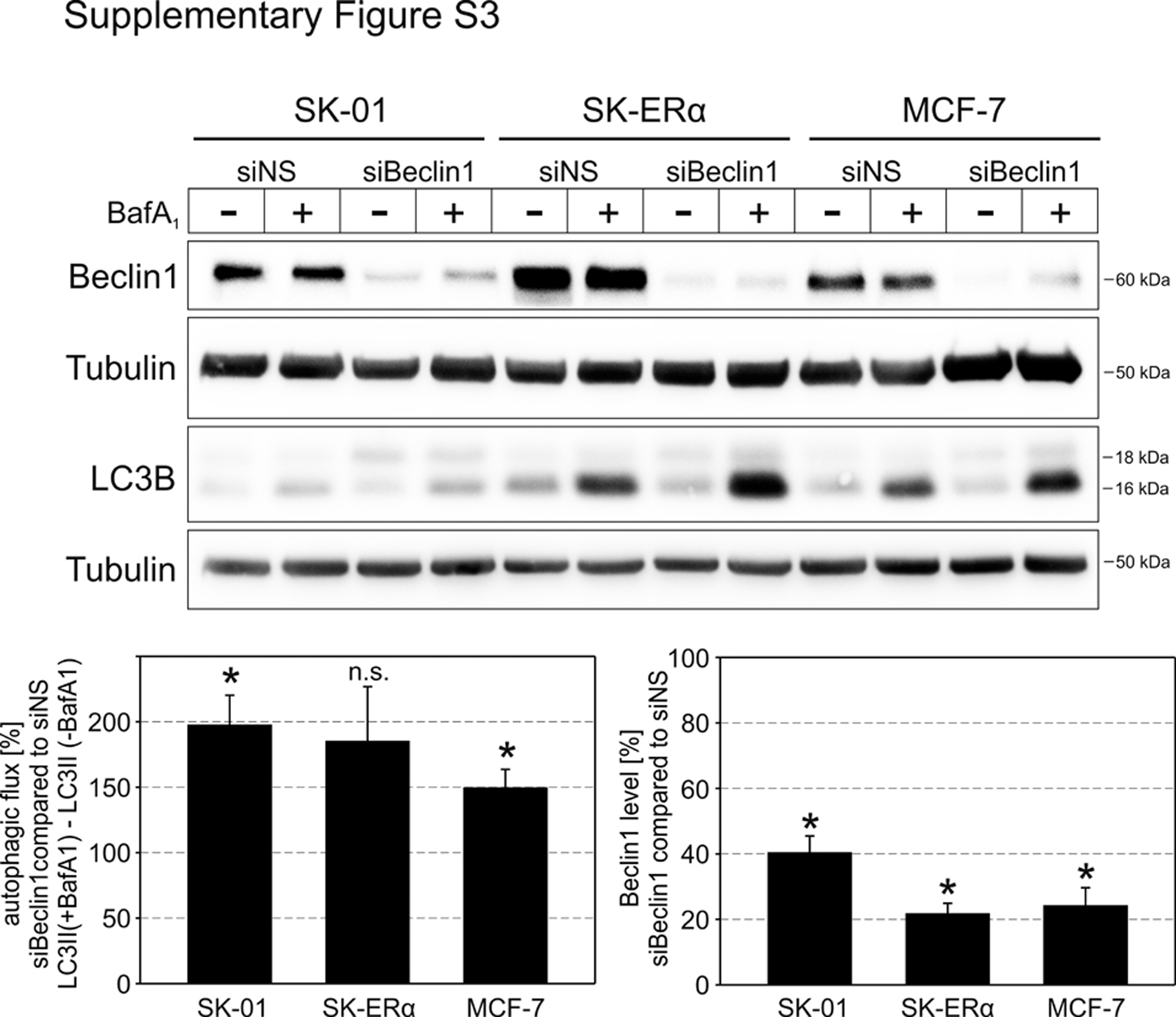

Supplement: Supplementary Figure 3 [file cddis2015181x4.tif]

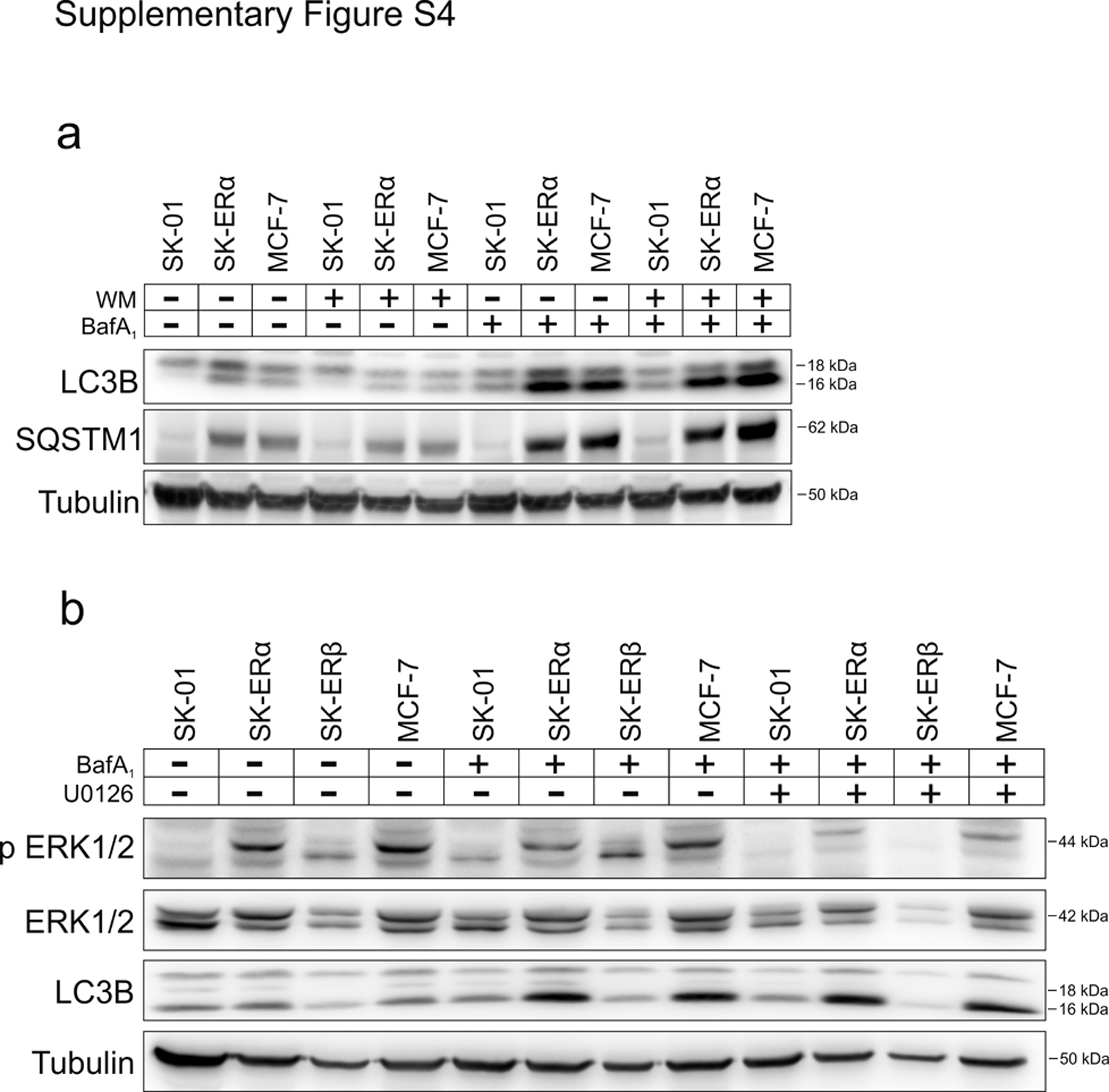

Supplement: Supplementary Figure 4 [file cddis2015181x5.tif]

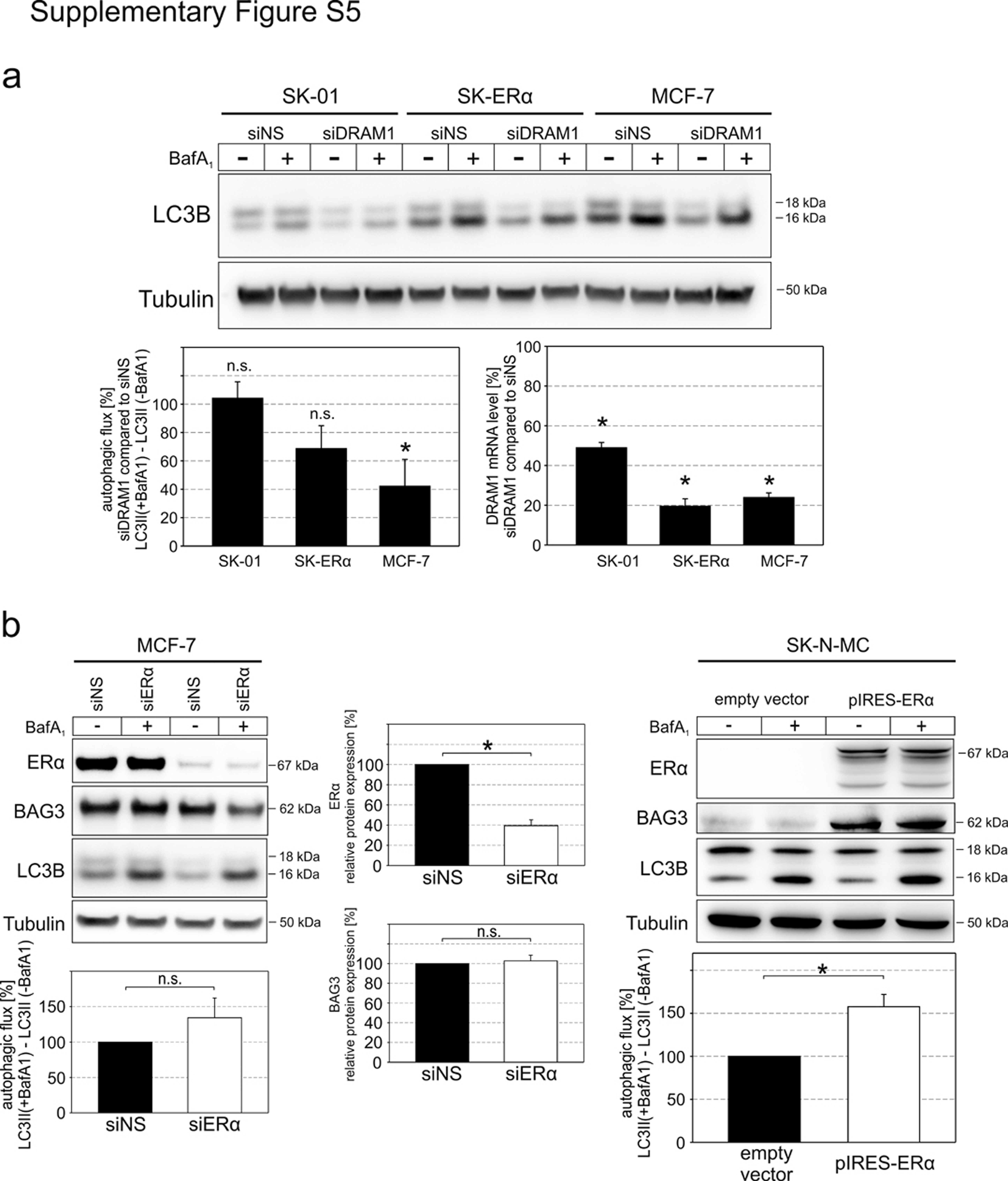

Supplement: Supplementary Figure 5 [file cddis2015181x6.tif]

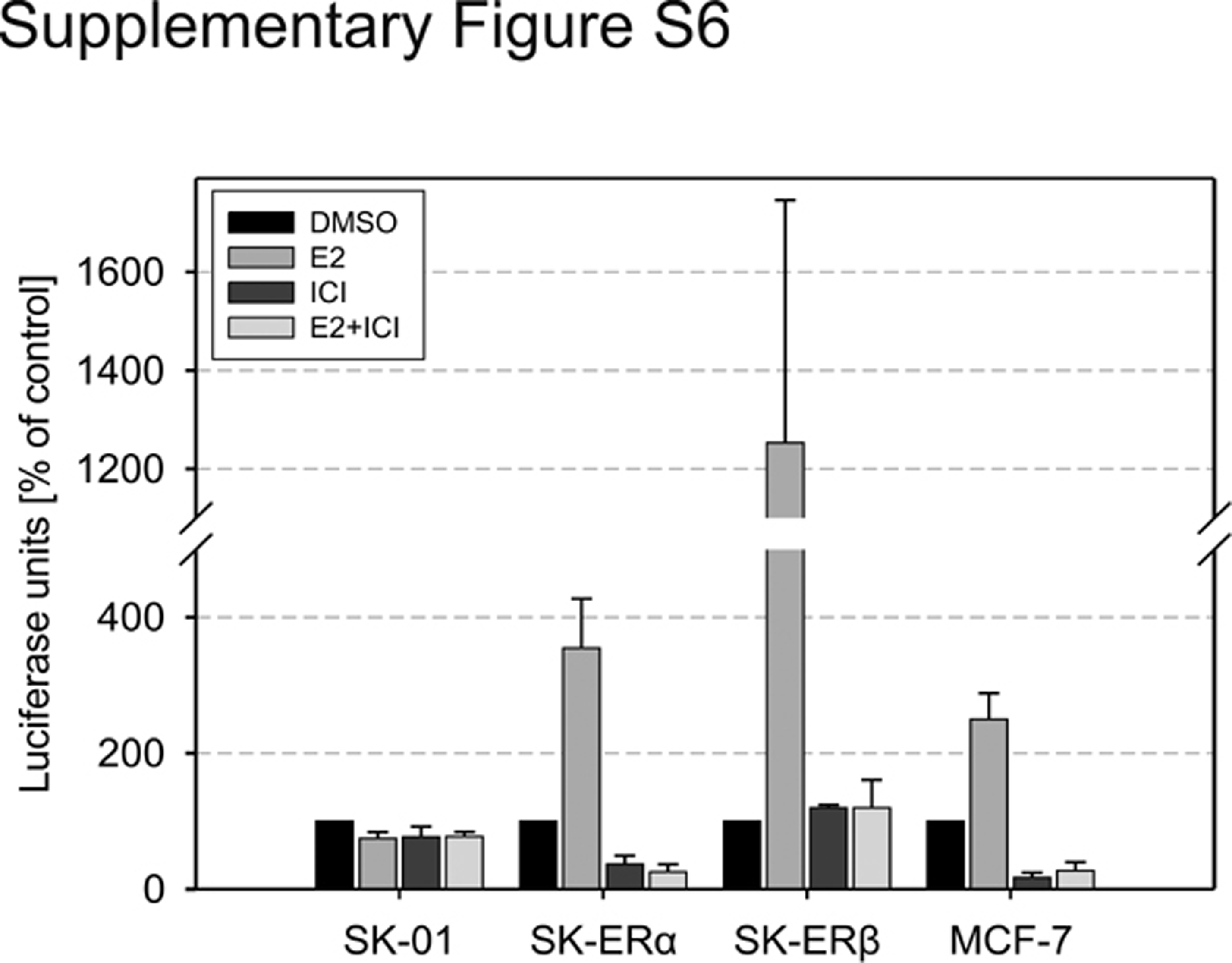

Supplement: Supplementary Figure 6 [file cddis2015181x7.tif]

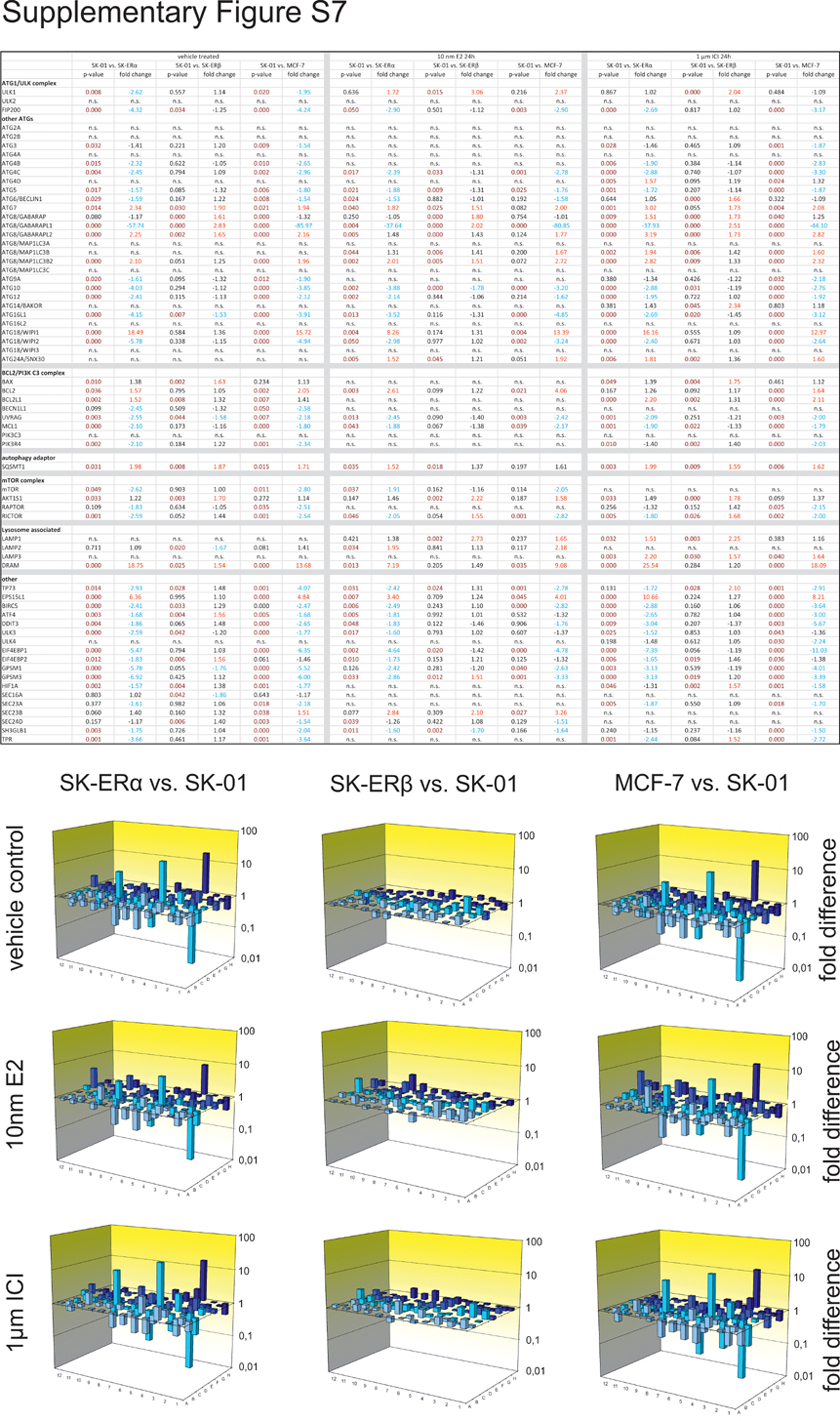

Supplement: Supplementary Figure 7 [file cddis2015181x8.tif]

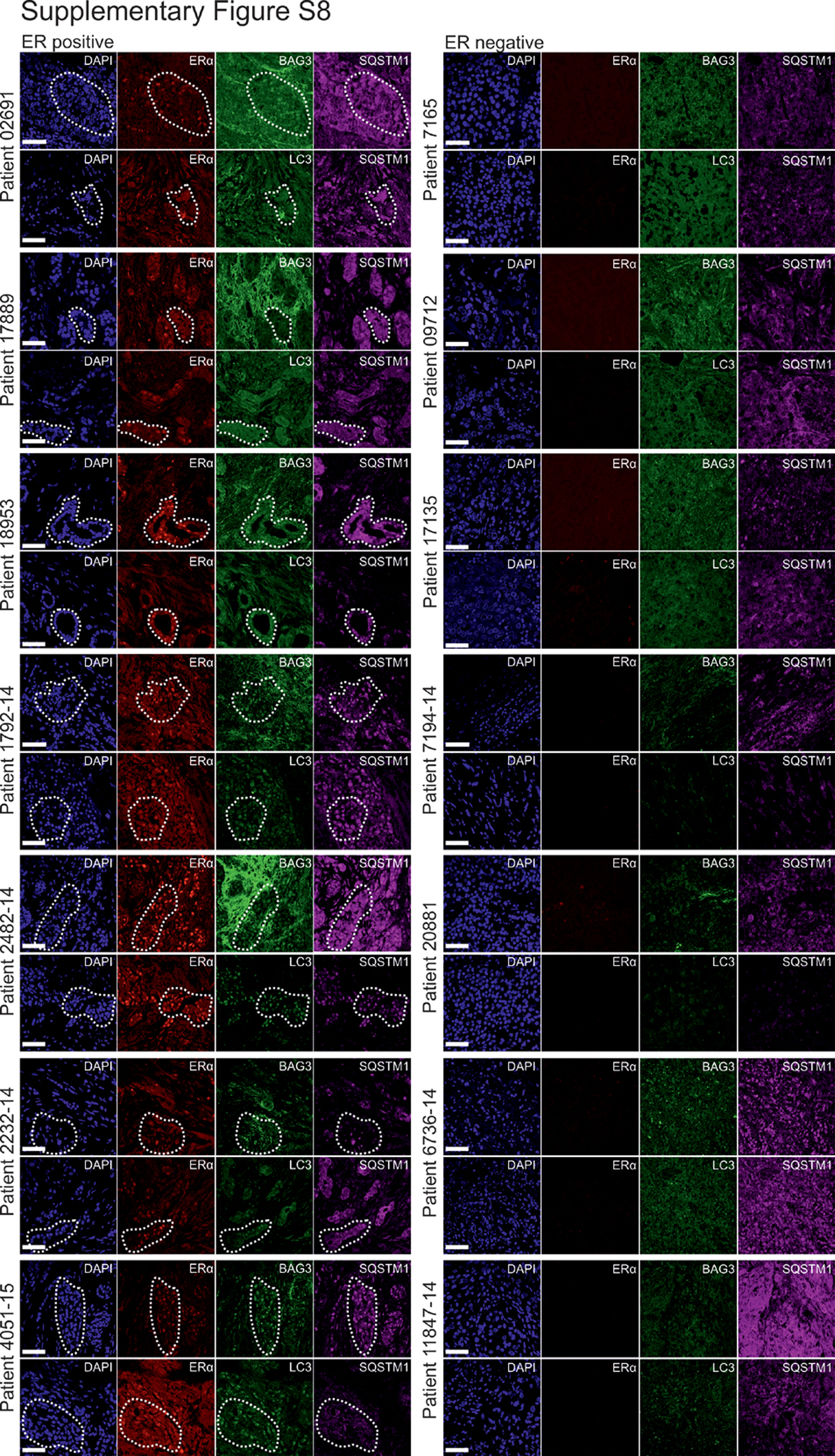

Supplement: Supplementary Figure 8 [file cddis2015181x9.tif]

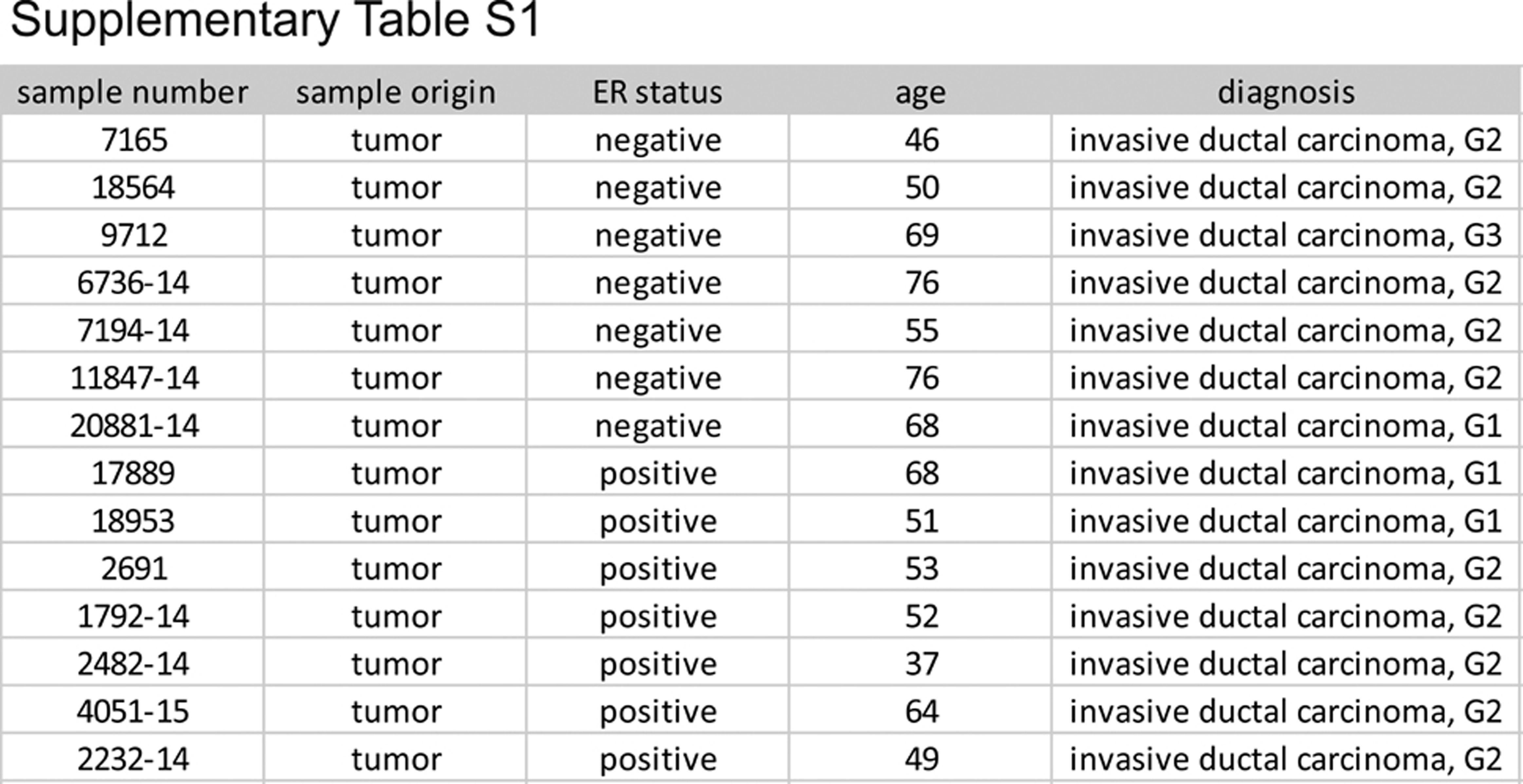

Supplement: Supplementary Table 1 [file cddis2015181x10.tif]
